# A novel mutation in intron 1 of *Wnt1* causes developmental loss of dopaminergic neurons in midbrain and ASD-like behaviors in rats

**DOI:** 10.1038/s41380-023-02223-8

**Published:** 2023-09-01

**Authors:** Yongyi Li, Mingwei Zhu, Wen-Xiong Chen, Jing Luo, Xin Li, Yangyang Cao, Meng Zheng, Shanshan Ma, Zhilan Xiao, Yani Zhang, Linyan Jiang, Xiumin Wang, Ting Tan, Xia Li, Qian Gong, Xiaoli Xiong, Jun Wang, Mingxi Tang, Mingtao Li, Ya-Ping Tang

**Affiliations:** 1 Guangdong Provincial Key Laboratory of Brain Function and Disease, Zhongshan School of Medicine, Sun Yat-sen University, Guangzhou, 510080 China; 2 Department of Pharmacology, Zhongshan School of Medicine, Sun Yat-sen University, Guangzhou, 510080 China; 3 Guangzhou Institute of Pediatrics, Guangzhou Women and Children’s Medical Center, Guangzhou Medical University, Guangzhou, 510623 China; 4 Department of Neurology, Guangzhou Women and Children’s Medical Center, Guangzhou Medical University, Guangzhou, 510623 China; 5 School of Basic Medicine, Southwest Medical University, Luzhou, Sichuan 646000 China; 6 Department of Pathology, Affiliated Hospital of Southwest Medical University, Luzhou, Sichuan 646000 China; 7 Department of Child Health, Third Affiliated Hospital of Zhengzhou University, Zhengzhou, Henan 450052 China

**Keywords:** Neuroscience, Molecular biology

## Abstract

Autism spectrum disorder (ASD) is a group of neurodevelopmental disorders with a strong genetic liability. Despite extensive studies, however, the underlying pathogenic mechanism still remains elusive. In the present study, we identified a homozygous mutation in the intron 1 of *Wnt1* via large-scale screening of ASD risk/causative genes and verified that this mutation created a new splicing donor site in the intron 1, and consequently, a decrease of WNT1 expression. Interestingly, humanized rat models harboring this mutation exhibited robust ASD-like behaviors including impaired ultrasonic vocalization (USV), decreased social interactions, and restricted and repetitive behaviors. Moreover, in the substantia nigra compacta (SNpc) and the ventral tegmental area (VTA) of mutant rats, dopaminergic (DAergic) neurons were dramatically lost, together with a comparable decrease in striatal DAergic fibers. Furthermore, using single-cell RNA sequencing, we demonstrated that the decreased DAergic neurons in these midbrain areas might attribute to a shift of the boundary of the local pool of progenitor cells from the hypothalamic floor plate to the midbrain floor plate during the early embryonic stage. Moreover, treatments of mutant rats with levodopa could attenuate the impaired USV and social interactions almost completely, but not the restricted and repetitive behaviors. Our results for the first time documented that the developmental loss of DAergic neurons in the midbrain underlies the pathogenesis of ASD, and that the abnormal progenitor cell patterning is a cellular underpinning for this developmental DAergic neuronal loss. Importantly, the effective dopamine therapy suggests a translational significance in the treatment of ASD.

## Introduction

Autism spectrum disorder (ASD, [DSM-5]) is a set of neurodevelopmentally defective conditions that are clinically characterized by impaired verbal communication and social interactions, and the expression of restricted and repetitive behaviors. Other than these core symptoms, ASD is often companied by many other mental comorbidities, such as anxiety, depression, learning disabilities, etc. [1]. At this moment, no effective medicine is available for the core symptoms. Recently, evidence from the animal studies as well as clinical trials has suggested that oxytocin might be effective, while it is still controversial [2]. Currently, the most effective therapies are behavior interventions. However, this type of therapy needs to be carried out at an early stage of age, usually before 6 years old, or even younger [3]. The problem is that an early diagnosis of ASD is representing a big challenge, and it is generally expected that this difficulty could be partially overcame by combining some other approaches together, such as risk gene tests [4] or brain imaging [5]. Therefore, to further investigate the underlying genetic, molecular, and neuronal mechanisms is not only important for a better understanding of the disease, but also of a high impact in our translational efforts on fighting against this disease, in terms of either a discovery of new diagnostic biomarkers or a development of new therapeutic strategies.

The genetic disposition of ASD was recognized early at the moment that the disease was reported, while the first line of the direct evidence came from family and twin studies, by which it has been found that the concordance rate of ASD is over 90%, around 46–67%, and up to 18%, respectively, among monozygotic twins, dizygotic twins, and full siblings [6, 7]. The genetic variants range from single nucleotide variants such as single nucleotide polymorphisms (SNPs) and point mutation, multiple-nucleotide or fragmental deletion or insertion, duplications, copy number variants (CNVs), and translocations to inversions etc. [8–10]. Thus, it is generally considered that the genetic factors for ASD are highly heterogeneous [4, 8, 10]. Indeed, if we just narrow down to variants in single genes, at least over 1000 risk genes have been identified so far [8, 11]. Many of those risk genes are related to transcriptional/translational activities, neuronal development, synaptic formation and functions, neurotransmissions, and molecular/signaling pathways etc.

WNT family of signaling molecules triggers at least three distinctive signaling cascades, among which the canonical WNT/ß-catenin signaling pathway plays an essential role in neuronal development and maintenance, such as cell proliferation and differentiation, neural stem cell or progenitor cell survival, neurogenesis, synaptic formation or function, neurotransmission etc. [12–15]. Evidence also indicates that the WNT/ß-catenin signaling pathway is particularly critical for the development of dopaminergic (DAergic) neurons in the brain [16]. It is now generally presumed that as a neuronal developmental disorder, ASD must endure certain disturbances in those neuronal or neural events or functions described above or others [17, 18]. Accordingly, any harmfully functional variants in gene(s) that encode for WNT itself or molecules within the signaling pathways may constitute an important genetic basis for ASD. Indeed, evidence is now available to support this notion. For example, the risk for ASD in people who harbors a *Wnt1* SNP (S88R) is 8-fold higher than that in people who does not have this SNP, since this SNP may lead to an over-activation of the WNT/ ß-catenin signaling [19]. In a Hmong family, two male siblings with ASD and severe intelligent deficit are both homozygous for a mutation of *Wnt1 c.884C*>A (p.Ser295*), which is predicted to produce a C-terminal truncated WNT1 protein [20]; and in another family, a female ASD proband harbors a homozygous mutation of *Wnt1* c.287-300del14, which is predicted to result in non-sense mediated mRNA decay and ultimately loss of WNT1 protein, while her parents and a brother are not ASD sufferers, since they are all heterozygous mutation only [20]. A reciprocal deletion or duplication of the 16p11.2 region is the most common CNVs that are associated with ASD, and the dysregulated signaling pathways implicated in this CNVs include WNT signaling [21]. All these findings indicate that at least three types of genetic variants, including SNPs, point mutation, and CNVs in *Wnt1* itself are significantly implicated in the pathogenesis of ASD. However, it is still not clear whether or how a mutant *Wnt1* triggers the pathogenesis of ASD.

Here, based on our clinical findings, we generated a humanized *Wnt1* mutant rat model, and our results demonstrated that this intron mutation is ASD-causative, and elucidated how a developmental loss of DAergic neurons in the midbrain constitutes to a pathogenic cascade for ASD, as well as how abnormal patterning of progenitor cells underlies this developmental neuronal loss. All of our results have provided with us new insights into the genetic and neuronal mechanisms underlying ASD, as well as into our translational efforts on fighting against ASD.

## Materials and methods

The details of the experimental materials and methodologies are given in the *SI Appendix*, including human blood samples, whole exome sequencing and sanger sequencing, animals, in vitro assay, rapid amplification of cDNA ends, chemical administration, behavioral tests, single-cell RNA sequencing and etc.

## Results

### A novel homozygous mutation in intron 1 of *Wnt1* causes a decrease of WNT1 expression

Based on our previous publication [22], we further expanded our samples from ASD patients. A novel homozygous mutation was found in *Wnt1* from an ASD female patient, but her parents were both harboring a heterozygous mutation and were both asymptomatic (Fig. S1A). The mutation changed the first nucleotide of the intron 1 from G to A (Fig. S1C) and was further confirmed by Sanger sequencing (Fig. S1B). Based on our analysis of the gene structure with the Ensemble, it was expected that this mutation would generate a new splice donor site at the intron 1. In order to test this speculation, we cloned human normal *Wnt1* and mutant *Wnt1* genomic DNA, namely *Wnt1*-nor-myc plasmid and *Wnt1*^sp^-myc plasmid, respectively, and then separately expressed in 293T cells. We determined the transcripts using 5′RACE, and the results were the same as what we expected from the Ensemble analysis, i.e., the first exon was lost while a fragment of 323 nt from the intron 1 was retained in the transcript (Figs. S2, S3A). Furthermore, an in vitro assay showed that a significant increase in the expression of myc tag and ß-catenin was found in *Wnt1*-nor-myc overexpression, but not in the *Wnt1*^sp^-myc overexpression (Fig. S3B–D), indicating that the mutation could cause the decrease of WNT1 expression, and decrease the activity of the canonical WNT/ ß-catenin signaling pathway as well.

### Generation of a humanized mutant rat model

In order to further explore whether this mutation was pathogenic or not, we generated a humanized mutant rat model that carried the same mutation by using CRISPR-Cas9 gene editing system. The introduced point mutation, from G to A (Fig. S4A), was confirmed by sequencing of the genomic DNA from mutant rats, and the genotypes were determined by a restriction enzyme (Hph1) digestion of PCR products from the tail genomic DNA of rats. Since the mutation created a new cutting site of Hph1, the PCR fragments were changed from 2 bands (wild-type, WT) to 3 bands (*Wnt1*^sp/+^) and 2 bands (*Wnt1*^sp/sp^) lower than WT (Fig. S4B). Rats including WT, heterozygous (*Wnt1*^sp/+^), and homozygous (*Wnt1*^sp/sp^) littermates were used in all experiments, unless stated elsewhere. With a 5′RACE, we further found that the mutation in rats, similarly to that in the patient, indeed created a new splicing site in the intron 1 of *Wnt1*, which led to the loss of the whole exon 1, but retainment of a fragment of 510 nt of the intron 1 (Fig. S5). With an in vitro overexpression system, we first confirmed the specificity of the WNT1 antibody (Fig. S6A), which consisting with the theoretical molecular weight and the signal that Myc antibody detected (Fig. S3B). Using Western blots, we then detected the expression level of WNT1 in the whole brain at different developmental stages, and the results showed that WNT1 was highly expressed in the brain at the embryonic day 12.5 (E12.5) and E15.5, but was decreased at postnatal day 7 (P7), P28, and P90 (Fig. S6B, C). The expression level of WNT1 tended to be stable both in WT (Fig. S6B, C) and *Wnt1*^*sp/sp*^ rats (Fig. S6D, E) after P7. Furthermore, we monitored the expression of WNT1 in different brain regions of the rats at P28, and the highest expression level was observed in the cerebellum, and then the hippocampus, striatum, forebrain, and a relatively lower level in the olfactory bulb, substantia nigra, and cortex (Fig. S6F, G). In addition, we found that the expression of WNT1 in *Wnt1*^*sp/sp*^ rats was decreased to 57.48%, compared to 88.57% in *Wnt1*^*sp/+*^, and 100% in WT rats (Fig. S6H, I). A significance difference was observed among WT, Wnt1sp/+, and *Wnt1*^*sp/sp*^ rats [F (2,7) = 21.55, *p* < 0.001], and *post-hoc* analysis revealed a significant difference between WT and Wnt1^sp/sp^ rats (p < 0.001) or between *Wnt1*^*sp/+*^ and *Wnt1*^*sp/sp*^ rats (*p* < 0.01), but not between WT and *Wnt1*^*sp/+*^ rats. These results indicate that this humanized rat model has been successfully generated, and it is indeed a WNT1 deficient model. The overall conditions of littermates of this model, including eating behavior, growth rate etc. were similar, while the body weight in *Wnt1*^sp/sp^ rats was significantly lower than that in WT rats (Fig. S7A). Although the brain weight of the *Wnt1*^sp/sp^ rats was slightly lower than that in WT rats (Fig. S7B), the histological changes at the gross level, including the organization of the brain regions and the shape etc. looked indistinguishable between WT and *Wnt1*^sp/sp^ rats (Fig. S7C, D), indicating that their overall condition was roughly similar.

### *Wnt1*^*sp/sp*^ rats exhibited robust ASD-like behaviors

**Fig. 1:**
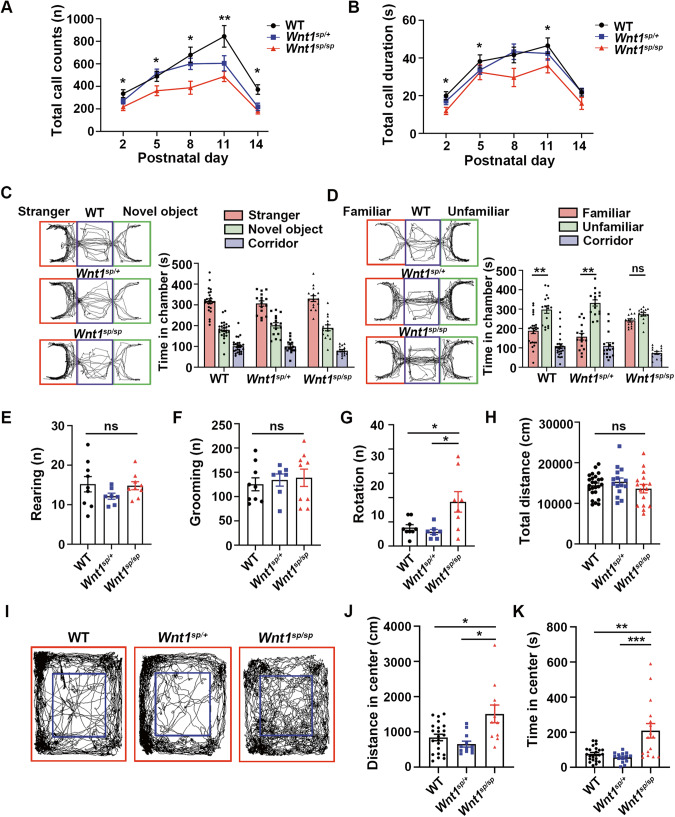
*Wnt1*^*sp/sp*^ rats exhibited robust ASD-like behaviors. **A**, **B** USV test. **A** Total call counts. **B** Total call duration. WT (*n* = 13–23), *Wnt1*^*sp/+*^ (*n* = 12–23), and *Wnt1*^*sp/sp*^ rats (*n* = 9–22) were examined. **C**, **D** Three-chamber test. **C** Social preference test. The left panel is the trajectory diagram, and the right bar figure shows the time spent in each chamber. **D** Social novelty test. The same as in (**C**). WT (*n* = 26), *Wnt1*^*sp/+*^ (*n* = 16), and *Wnt1*^*sp/sp*^ (*n* = 14) were examined. **E**–**K** Open-field test. **E** Number of rearing. **F** Number of grooming. **G** Number of rotation. **H** Total distance traveled. **I** Trajectory diagram of open-field test: the central area (blue square) and the peripheral area (the other parts). **J** Distance traveled in the central area. WT (*n* = 22), *Wnt1*^*sp/+*^ (*n* = 14), and *Wnt1*^*sp/sp*^ rats (*n* = 16) were examined. **K** Time spent in the central area. WT (*n* = 22), *Wnt1*^*sp/+*^ (*n* = 14), and *Wnt1*^*sp/sp*^ (*n* = 16) were examined. All data are expressed as mean ± SEM. *p < 0.05; **p < 0.01; ***p < 0.001, one-way ANOVA followed by Duncan’s test.

To determine whether this novel mutation was pathogenic or not, we examined the mutant rats with a battery of behavioral tests that are commonly used in ASD modeling in the rodents. The first one was an ultrasonic vocalization (USV) test. Rats perceive and emit calls in an ultrasonic range, and this vocalization is generally considered as a “communication” among them. Typically, three paradigms, isolation-induced USV in pups, interaction-induced USV in juveniles, and interaction-induced USV in adults, can be a choice [23]. Here, we employed the first one. The total call counts (Fig. 1A) and call duration (Fig. 1B) were both increased from P2 to P11, but decreased from P11 to P14, indicating that all these rats had a similar pattern of vocalization. However, an one-way ANOVA revealed a significant difference in the total call counts at P2 [*F* (2,55) = 6.299, *p* < 0.05], P5 [*F* (2,60) = 4.227, *p* < 0.05], P8 [*F* (2,59) = 5.851, *p* < 0.01], P11 [*F* (2,52) = 5.608, *p* < 0.01], P14 [*F* (2,59) = 3.313, *p* < 0.05] or in the call duration at P2 [*F* (2,58) = 3.982, *p* < 0.05], P5 [F (2,58) = 4.327, *p* < 0.05], but not P8 [*F* (2,59) = 2.832, *p* > 0.05], P11 [*F* (2,52) = 5.533, *p* < 0.05], but not P14 [*F* (2,55) = 0.162, *p* > 0.05]. Detailed *post-hoc* analyses with Duncan’s test revealed that a significant difference (*p* < 0.05–0.001) was observed in the call counts in all tested ages, or in the call duration in most ages tested (except for P8 and P14) between WT and *Wnt1*^sp/sp^ rats, or between *Wnt1*^sp/+^ and *Wnt1*^sp/sp^ rats, but not between WT and *Wnt1*^sp/+^ rats. All these results indicated that the mutation at the homozygous condition could significantly impair the “communication” in these rats.

Then, a three-chamber test was used. This test detects a social preference and a social novelty in the rodents via comparing the time spent in confronting a stranger vs confronting an inanimate novel object, or confronting an unfamiliar vs confronting a familiar rat, respectively. In the social preference test (Fig. 1C, left panel), all rats in three genotypes spent a similar duration of time in chamber containing a stranger (Fig. 1C, right bar figure), indicating that the social preference in all these rats was at a similar level. In the social novelty test (Fig. 1D, left panel), however, a significantly longer duration of time in the chamber containing an unfamiliar rat than in the chamber containing a familiar one was noted in either WT [*F* (2,75) = 40.92, *p* < 0.001] or *Wnt1*^sp/+^ rats [*F* (2,45) = 49.490, *p* < 0.001], but not in *Wnt1*^*sp/sp*^ rats. Instead, a significant difference was observed between WT and *Wnt1*^*sp/sp*^ rats (*p* < 0.05), but not between WT and *Wnt1*^sp/+^ rats (Fig. 1D), indicating that the mutation at the homozygous condition could impair the social novelty significantly.

ASD rodent model may express spontaneous stereotypic behaviors, such as rotation, jumping, rearing, and self-grooming, which can be evaluated with an open-field test. Although a significant difference in rearing (Fig. 1E) or grooming (Fig. 1F) was not noted between any two groups of rats examined, *Wnt1*^sp/sp^ rats showed a significantly higher level in the rotation behavior [*F* (2,21) = 6.394, *p* < 0.05] (Fig. 1G). Detailed *post-hoc* analyses revealed a significant difference between WT and *Wnt1*^sp/sp^ rats (*p* < 0.05), and between *Wnt1*^sp/+^ and *Wnt1*^sp/sp^ rats (*p* < 0.05), but not between WT and *Wnt1*^sp/+^ rats, indicating that the mutation at the homozygous condition could lead to certain degree of repetitive behavior. In non-stereotypical behavioral observation (Fig. 1I), a significant difference in the total distance traveled was not found between any two groups of rats examined (Fig. 1H). However, *Wnt1*^*sp/sp*^ rats spent significantly more time [*F* (2,53) = 9.638, *p* < 0.001] (Fig. 1J), or traveled significantly longer distance [*F* (2,42) = 8.63, *p* < 0.001] (Fig. 1K) in the central area than those of WT or *Wnt1*^sp/+^ rats did. Detailed *post-hoc* analyses revealed a significant difference between WT and *Wnt1*^sp/sp^ rats in the distance traveled (*p* < 0.05) or time spent (*p* < 0.01) in the central area, or between *Wnt1*^sp/+^ and *Wnt1*^sp/sp^ rats in the distance traveled (*p* < 0.05) or time spent (*p* < 0.001) in the central area, but not between WT and *Wnt1*^sp/+^ rats, suggesting that the mutation at the homozygous condition might lead to abnormal behaviors in confront to a conflict environment.

In conclusion, all these results above indicated that the humanized mutant rats exhibited robust ASD-like behaviors in three behavioral paradigms.

### *Wnt1*^*sp/sp*^ rats showed developmental loss of DAergic neurons in the midbrain

**Fig. 2:**
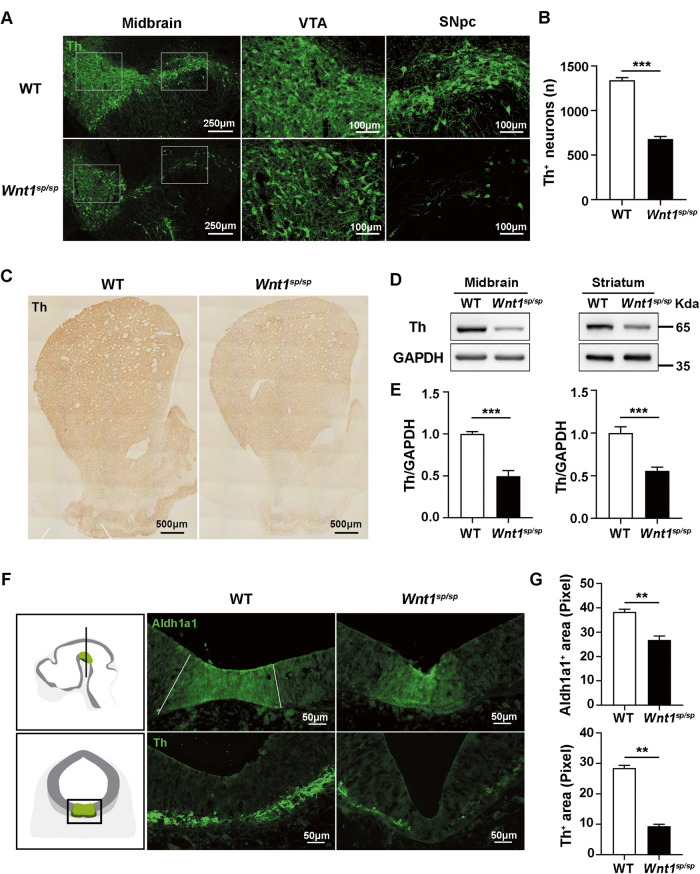
Developmental loss of DAergic neurons in *Wnt1*^*sp/sp*^ rats. **A** Th immunofluorescence staining of brain sections of WT (*n* = 3) and *Wnt1*^*sp/sp*^ rats (*n* = 3) at P28. **B** Quantitative analyses of (**A**). **C** Th immunohistochemical staining of the striatum in WT (*n* = 3) and *Wnt1*^*sp/sp*^ rats (*n* = 3) at P28. **D** Western blot shows the expression of Th in the midbrain (*n* = 10) and striatum (*n* = 7). **E** Quantitative analyses of (**D**). **F** The left panel (from Baless and Ang [56]) shows the position of the coronal section as well as where the picture is zoomed in the right panel. The immunofluorescence staining of brain sections of WT (*n* = 3) and *Wnt*^*sp/sp*^*Wnt1*^*sp/sp*^ rats (*n* = 3) at E12.5 with Aldh1a1 or Th. **G** Quantitative analyses of the staining signals in (**F**). All data are expressed as mean ± SEM. **p < 0.01; ***p < 0.001, Student’s *t* test.

WNT signaling plays an important role in neuronal development, especially for DAergic neurons. However, at the gross anatomical level, we did not find an obvious difference between WT and *Wnt1*^*sp/sp*^ rats (Fig. S7C, D), which was different from that reported in *Wnt1* knockout (KO) mice, in which an obvious shrinkage of the midbrain was found [24]. Therefore, we needed to answer whether, at a fine anatomical level, there was any difference between WT and *Wnt1*^*sp/sp*^ rats. Accordingly, Aldh1a1 and Th were respectively used to label precursor cells and mature DAergic neurons. To our surprising, the number of Th^+^ DAergic neurons in the substantia nigra compacta (SNpc) and the ventral tegmental area (VTA) regions together was significantly decreased by about 46% in the midbrain of *Wnt1*^*sp/sp*^ rats at P28, compared to that in WT littermates (Fig. 2A, B). Correspondingly, the DAergic fibers projected to the striatum were also diminished (Fig. 2C). More consistently, the expression of Th in the midbrain and the striatum of *Wnt1*^*sp/sp*^ rats was decreased by 49.80% and 55.87%, compared to that in WT littermates, respectively (Fig. 2D, E). However, we found that the number of the Th^+^ DAergic neurons in *Wnt1*^*sp/sp*^ rats was decreased by 47.95%, 50.84%, and 54.79% at P7, P28 and P90, respectively (Fig. S8A–C), suggesting that these DAergic neurons didn’t go through a progressive loss following aging. We further detected the expression level of Th at P7, P28, and P90, and the results consistently showed a similar changed pattern as described above (Fig. S8E–G). At the initial stage of DAergic neuronal differentiation (E12.5), both precursor cells and DAergic neurons were decreased in *Wnt1*^*sp/sp*^ rats dramatically (Fig. 2F, G). What’s more, a developmental loss of the Th^+^ neurons was detected from E15.5 to P7 (Fig. S8G). All these results suggested that the developmental loss of DAergic neurons in the midbrain of *Wnt1*^*sp/sp*^ rats was due to a decreased resource for the differentiation of DAergic neurons, and this reduction lasted into an after birth-life stage at a relatively stable level (about 50%).

### *Wnt1*^*sp/sp*^ rats exhibited a changed patterning of progenitor cells in two key brain subregions

The midbrain/hindbrain floor plate (MHFP) and ZLI-hypothalamic floor plate (ZHFP) are neighbored together, and a normal patterning of progenitor cells in these two brain subregions is critical for the subsequential distribution of a specific neuronal type, such as DAergic neurons and glutaminergic neurons etc. in its prearranged brain regional manner. So, it is of a fundamental interest to determine if the developmental loss of DAergic neurons in our model is due to an abnormal patterning of progenitor cells. Accordingly, we conducted single-cell RNA sequencing (scRNA-seq). The poly-A RNA from brain tissues containing the diencephalon, midbrain, hindbrain, but not the forebrain (Fig. S9A) was extracted at the age of embryonic day 11.5 (E11.5), and then was subjected to droplet-based scRNA-seq. A total of 11 cell clusters could be identified, among which the largest one was neural progenitor cells (Fig. S9B). These cells were visualized by four markers including Sox2, Rfx4, Adgrv1, and Fat3 (Fig. S9C), and were named as neural tube organizers (NTO) [25]. These cells could be further sub-divided into 6 subclusters (Fig. S9D), and a spatial pattern of these cells was observed in the floor plate (FP), diencephalic roof plate, hindbrain roof plate, isthmus, basal/alar plate and unknown neuronal population (Fig. S9E). All these results established the unique technical basis for a further study of these progenitor cells in rats.

**Fig. 3:**
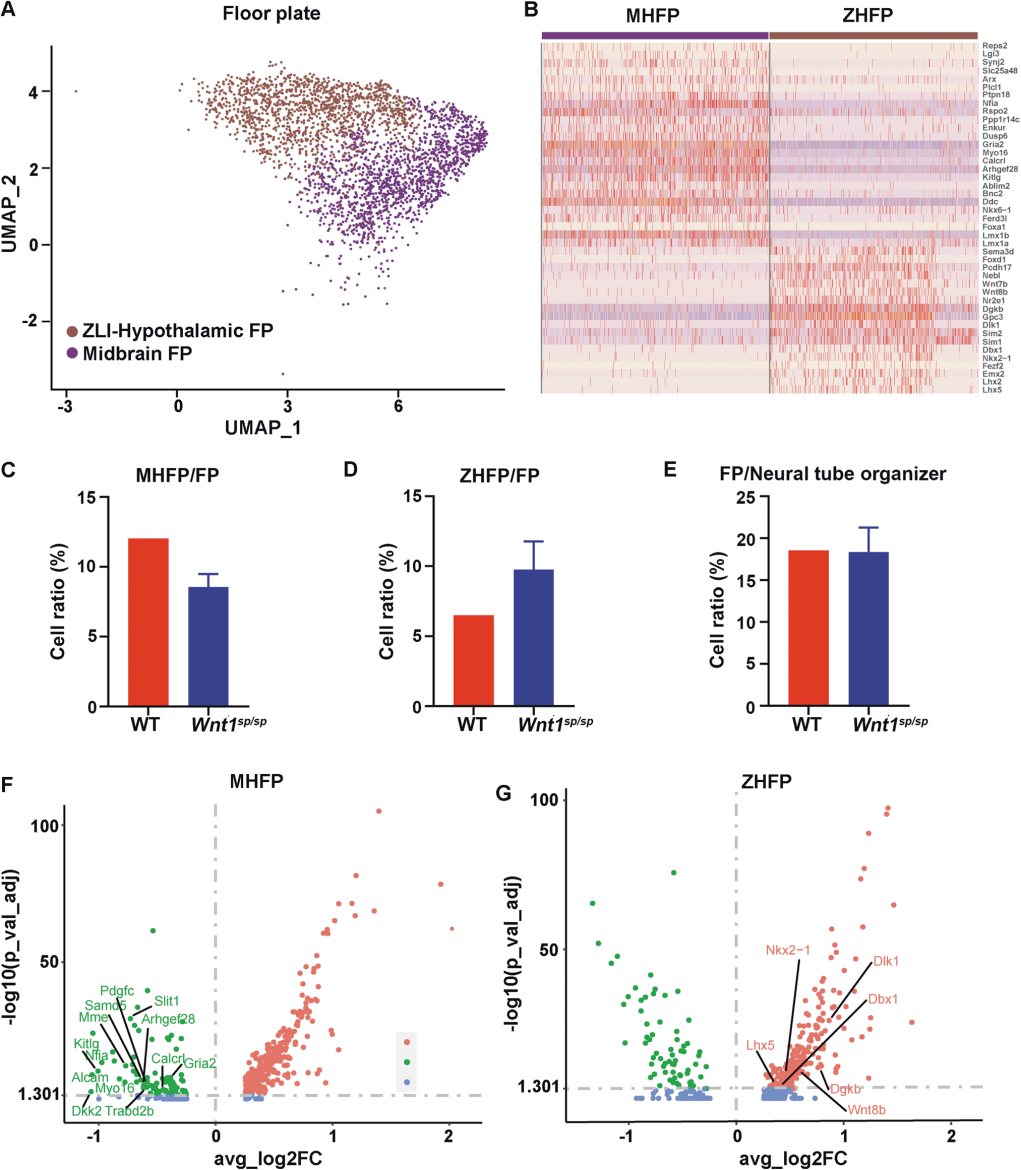
Single-cell RNA sequencing revealed a changed patterning of progenitor cells in two key brain subregions in *Wnt1*^*sp/sp*^ rats. **A** UMAP map of 2 subclusters of the FP. **B** Heat map of the expression of marker genes for the MHFP or the ZHFP. **C** Ratio of progenitor cells in the MHFP/entire FP. **D** Ratio of progenitor cells in the ZHFP/entire FP. **E** Ratio of progenitor cells in the FP/neural tube organizer. **F** The volcano map of differential expression of genes including down-regulation (green), upregulation (pink), or no change (blue) in the MHFP cluster. **G** The same volcano map in the ZHFP cluster.

**Fig. 4:**
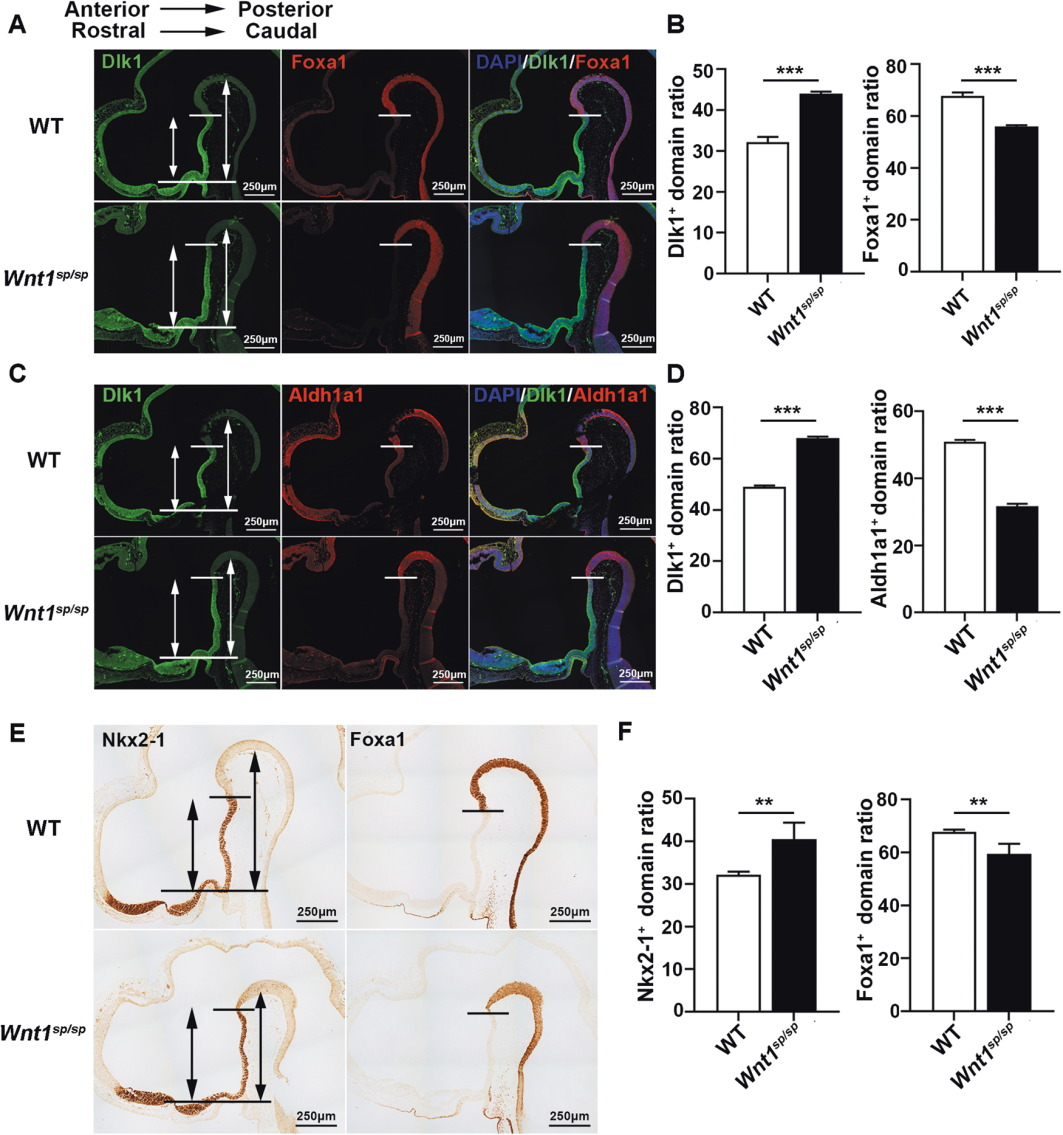
*Wnt1*^*sp/sp*^ rats display an abnormal patterning of progenitor cells between MHFP and ZHFP. **A** Double staining with Dlk1 (green) and Foxa1 (red) in WT and *Wnt1*^*sp/sp*^ rats at E12.5. Overlapped signaling shows the boundary between the MHFP and ZHFP that moves to the MHFP. **B** Quantitative analyses of (**A**). **C** Double staining with Dlk1 and Aldh1a1 in WT and *Wnt1*^*sp/sp*^ rats at E12.5. Overlapped signaling shows the boundary between ZHFP and MHFP moves to the MHFP. **D** Quantitative analyses of (**C**). **E** Immunohistochemical staining of Nkx2-1 and Foxa1 on adjacent sections of the brain from WT and *Wnt1*^*sp/sp*^ rats at E12.5; **F** Quantitative analyses of (**E**). Short line represents the boundary, long line represents the rostral end of ventral FP. The short double arrow line represents the length of ZHFP and the long double arrow line represents the half-length of the whole FP. Three rats in each group, and data are expressed as mean ± SEM. *p < 0.05, Student’s *t* test.

As DAergic neurons are thought to be originated from the FP of the neural tube [26], a further study of the FP cluster is important. Based on the expression profiling of their markers, we could further divide the FP into two subclusters (Fig. 3A), i.e. the MHFP, which specifically expressed Lmx1a, Lmx1b, Foxa1, Ferd3l, Ddc, etc. (Fig. 3B), and the ZHFP, which specifically expressed Lhx5, Lhx2, Emx2, Fezf2, Nkx2-1, Dbx1, Sim1, Sim2, Dlk1, Gpc3, Dgkb, etc. (Fig. 3B). Surprisingly, the ratio of the progenitor cells in the MHFP over the entire FP in *Wnt1*^*sp/sp*^ rats (8.3%) was dramatically lower than that in WT rats (12.0%) (Fig. 3C), and the ratio in the ZHFP over the entire FP in *Wnt1*^*sp/sp*^ rats (9.4%) was significantly higher than that in WT rats (6.5%) (Fig. 3D). However, the cell ratio in the FP over the total number of 6 cluster (NTO) in *Wnt1*^*sp/sp*^ rats (17.8%) was very similar to that in WT rats (18.5%) (Fig. 3E). Moreover, the expression level of the marker genes for these progenitor cells in the MHFP in *Wnt1*^*sp/sp*^ rats including Arhgef28, Dkk2, Kitlg, Alcam, Myo16, Samd5, Calcrl, and Gprc5c, was significantly lower than those in WT rats (Fig. 3F), but the expression level of the marker genes for the progenitor cells in the ZHFP in *Wnt1*^*sp/sp*^ rats including Gpc3, Dlk1, Dgkb, Wnt8b, Nkx2-1, Lhx5, and Dbx1, was significantly higher than those in WT rats (Fig. 3G). We further used RT-qPCR to verify the expression level of all these markers. As shown in Fig. S10, the expression of the markers for the MHFP was dramatically downregulated (Fig. S10A), while the expression of the markers for the ZHFP was dramatically upregulated (Fig. S10B). Based on their specificity (Fig. S10C, D) and the expression level, we further choose Foxa1 as a key marker for the MHFP and Dlk1 and Nkx2-1 for the ZHFP. Consistently, Western blot showed a lower expression level for Foxa1 (Fig. S10E, F), but a higher level for both Dlk1 (Fig. S10G, H) and Nkx2-1 (Fig. S10I, J) in *Wnt1*^*sp/sp*^ rats, compared respectively to that in WT rats. Furthermore, we conducted immunohistochemistry and immunofluorescence to stain two respective markers in a combination of Dlk1 and Foxa1 (Fig. 4A, B), or Dlk1 and Aldh1a1 (Fig. 4C, D), or Nkx2-1 and Foxa1 (Fig. 4E, F). The results showed that the boundary between the expression of the two markers in every combination was shifted from the ZHFP to the MHFP. All these results at four levels strongly suggested that there was a significant loss of progenitor cells in the MHFP.

Taken together, all these results indicated that the developmental loss of DAergic neurons in our *Wnt1*^*sp/sp*^ rats might attribute to the changes of the patterning of the progenitor cells between these two critical brain subregions.

### DA replacement therapy (DA-RT) largely rescued ASD-like behaviors of *Wnt1*^*sp/sp*^ rats

**Fig. 5:**
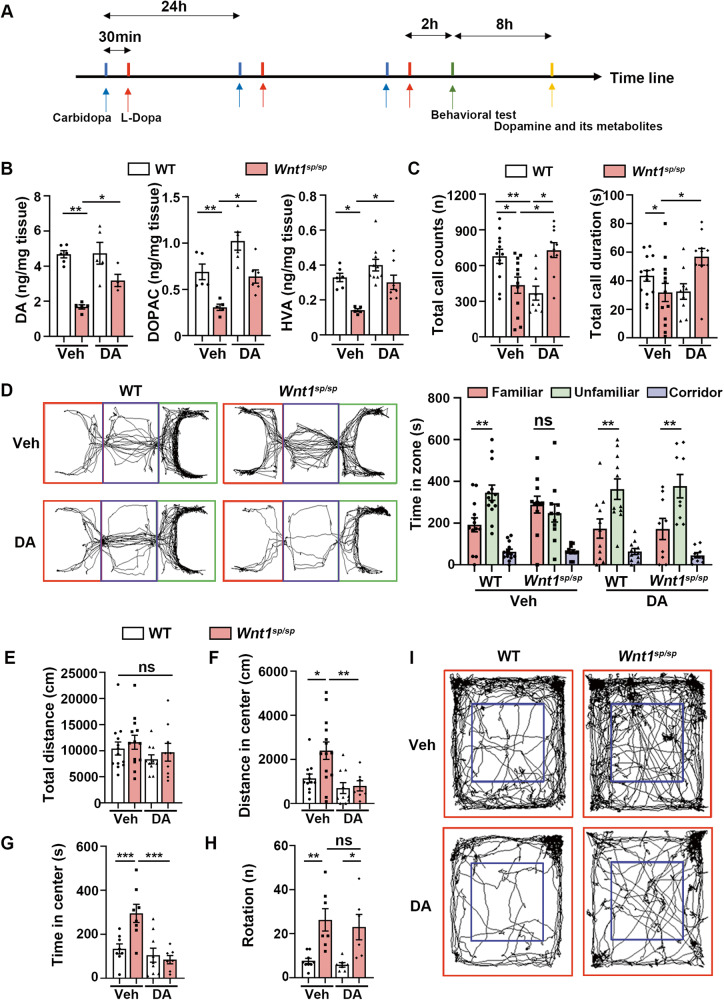
DA replacement therapy (DA-RT) largely rescued ASD-like behaviors in *Wnt1*^*sp/sp*^ rats. **A** Schematic paradigm for DA-RT. **B** DA and its metabolites in the striatum (*n* = 5–10). **C** Total call counts and call duration in USV test. WT (*n* = 9–13) and *Wnt1*^*sp/sp*^ (*n* = 8–13) rats at P11 were used. **D** Left panel is the trajectory diagram, and right bar figure shows the time spent in each chamber. WT (*n* = 11–13) and *Wnt1*^*sp/sp*^ rats (*n* = 7–11) were examined. **E**–**I** Open-field test. **E** Total distance traveled. **F** Distance traveled in the central area. **G** Time spent in the central area. **H** Number of rotations. **I** Trajectory diagram of the test: the central area (blue square) and the peripheral area (the other parts). WT (*n* = 7–12) and *Wnt1*^*sp/sp*^ rats (*n* = 7–13) were examined. Data are expressed as mean ± SEM. *p < 0.05; **p < 0.01; ***p < 0.001, Student’s *t* test.

Whether the developmental loss of DAergic neurons caused the ASD-like behaviors was still not clear. Given the fact that the functional disturbance of loss of DAergic neurons could be largely rescued by DA-RT, we designed a paradigm as shown in Fig. 5A. We confirmed that this paradigm effectively increased the striatal DA level in *Wnt1*^*sp/sp*^ rats (Fig. 5B) by using a high-performance liquid chromatography-mass spectrometry (HPLC-MS). In the USV test, the total call counts and call duration in *Wnt1*^*sp/sp*^ rats received DA-RT were both returned to the level in WT rats (Fig. 5C). In the three-chamber test, no significant difference in social preference was observed between any two groups of rats after the DA-RT (Fig. S11A). In the social novelty test, however, *Wnt1*^*sp/sp*^ rats received DA-RT spent a significantly longer duration of time in the chamber containing an unfamiliar rat than in chamber containing the familiar one (Fig. 5D). Lastly, in the open-field test, although the DA-RT in *Wnt1*^*sp/sp*^ rats did not produce an obvious change in the total travel distance (Fig. 5E), it significantly decreased the travel distance and time spent in the central area (Fig. 5F, G, I). As shown in Fig. 5H, however, there was no significant difference in rotation behavior between *Wnt1*^*sp/sp*^ rats treated with levodopa and *Wnt1*^*sp/sp*^ rats treated with vehicle, indicating that the DA-RT could not restored the restricted and repetitive-like behaviors. It is worth to mention that the short-term DA-RT did not increase the motor activity of mutant rats, since the total distance rats traveled in open filed and three chamber was not changed as well as the travel speed in open field (Fig. 5E, Fig. S11B, C). All the results indicated that the DA-RT could largely rescue the ASD-like behaviors in our ASD rat model.

## Discussion

In this study, we identified a novel mutation in the intron 1 of *Wnt1*. With our humanized mutant rat model, we for the first time demonstrated that this mutation could lead to the developmental loss of DAergic neurons in the midbrain, and subsequently, ASD-like behaviors. Most importantly, we found that the abnormal patterning of progenitor cells between the MHFP and ZHFP might be the underlying mechanism for the developmental loss of DAergic neurons, and DA-RT is effective for most of the ASD-like behaviors.

It should be noted that it is not new to state that a dysfunctional WNT signaling pathway may be pathogenically involved in ASD, since mutations in either *Wnt1* itself or in the molecules that encompass this signaling pathway were frequently reported to be associated with ASD [19–21]. In our case, however, there are two unique features. First, this is the first time to identify a new mutation in the intron 1. We, at three levels, bioinformatics, in vitro and in vivo experiments, verified that this intron mutation could create a new splicing site, and as a consequence, the expression of WNT1 was decreased. At the same time, the canonical WNT/ß-catenin signaling pathway was inactivated at the in vitro level (Fig. S3B–D). In vivo, although no change was detected in the ß-catenin expression in *Wnt1*^*sp/sp*^ rats (Fig. S12A), a decreased expression level of its target gene, Otx2 [27] (Fig. S12B–F), was found, suggesting that the WNT/ß-catenin signaling was inactivated. Conclusively, we demonstrated a loss-of-function mechanism for this intron mutation. Second, the mutant rats showed robust ASD-like behaviors. Although some of early reports showed an “association” of *Wnt1* mutation with ASD [20, 28], there were no any functional studies so far to confirm whether those mutations directly cause ASD or not, and thus, those clinical associations just stand for a sort of speculation only. In our study, we employed three classical behavioral paradigms to evaluate the ASD-like core symptoms, and importantly, *Wnt1*^*sp/sp*^ rats consistently show ASD-like behaviors in all these three tests, confirming the pathogenic role of this homozygous mutation. Our report thus for the first time demonstrated that the *Wnt1* mutation harboring a loss-of-function mechanism is ASD-causative.

The reasons for why we could not detect any changes in ß-catenin in vivo might be due to (1) Only a small portion of progenitor cells is responded to the WNT1/ß-catenin signaling. Firstly, the scRNA-Seq result in this study and other studies all demonstrated that WNT1 expression was regionally specific. It was highly expressed in the midbrain floor plate and the hindbrain roof plate progenitor cells (Fig. S13A, B) rather than expressed broadly. Thus, WNT1 deficiency is likely to occur only in these portions of cells. Secondly, a report conducted on BAT-gal reporter mice found that (data showed in its Supplemental materials) the number of DAergic progenitor cells in response to ß-catenin (Pitx3^+^/ß-GAL^+^) was decreased following *Wnt1* knockout, but the number of these cells (Pitx3^+^/ß-GAL^+^) accounts only for a very low proportion of the total number of ß-GAL^+^ cells [29]. Therefore, Western blot with bulk embryonic brain tissue may not be sufficient to detect such minor changes; (2) There may be a compensatory activation of WNT/ß-catenin signaling pathway. Dkk family is the upstream inhibitor while R-spondin family is the upstream activator of the WNT signaling pathway respectively [30]. In our study, scRNA-seq result showed that both Dkk2 and Rspo2 are highly expressed in FP (Fig. S13C, D) where WNT1 is highly expressed as well. Further, scRNA-seq (Fig. S13E) combined with RT-qPCR (Fig. S13F, G) data showed that Dkk2 was downregulated and Rspo2 was upregulated in *Wnt1*^*sp/sp*^ rats compared with WT rats, they may together compensate the expression or activity of ß-catenin; (3) The time window for WNT1/ß-catenin signaling pathway promoting neuronal development is earlier than E12.5. This notion was supported by a study which found that the effect that WNT1 contributed to the development of midbrain DAergic neurons peaked at E9.5 and E11.5, but ceased at E13.5 [31]. Unfortunately, E11.5–E12.5 rat embryos were the earliest embryos available for us.

Actually, given that ASD is a neuronal developmental disorder, and that WNT signaling plays an important role in neuronal development [12] and functions [13–15], it should not be so surprising to demonstrate the pathological role of *Wnt1* mutation in ASD. In terms of homozygous mutation, however, clinical findings were not always consistent. For example, two female siblings who were both homozygous for *Wnt1* c.884C > A mutation were diagnosed differently, the younger one was ASD, while the elder one was not [32]. Those clinical findings seem to not completely consist with our findings, the nature of this mutation may be different from that we found in *Wnt1*^*sp/sp*^ mutation. As described above, in our case, the *Wnt1* mutation leads to the decreased expression of WNT1 protein both in vitro and in vivo, while *Wnt1* c.884C > A (p. Ser295*) was confirmed to produce C-terminal truncated WNT1 and could not increase the activated form of ß-catenin (non-p-ß-catenin) compare with normal WNT1. Furthermore, it led to an increased expression level of WNT1 protein level in vitro as well [33]. But the effect of this mutation under the in vivo condition was not clear. The deleterious effect of the mutation in our study may be more serious. We could not exclude the possibility that the onset of ASD in their cases might be not purely dependent on the genetic effect, but is also related to some other factors, such as environmental factors [34]. It is worth to mention that we didn’t find any ASD phenotypes in *Wnt1*^*sp/+*^ rats, which consistent with the condition of parents in the pedigree of this study. This phenomenon may be attributed to the fact that *Wnt1*^*sp/+*^ mutation neither resulted in a significant decrease in WNT1 protein level (Fig. S6H, I) nor DAergic neurons (Fig. S8A, D, E, H). Abnormality in WNT signaling pathways is associated with a variety of neurological disorders, including schizophrenia (SCZ) [35]. It is worth to note that the clinical symptoms in deficit-SCZ include an impaired social interaction, diminished motivation, reduced talking, depression and anxiety, which are similar to those observed in certain cases of ASD [36]. However, in this study, *Wnt1*^*sp/sp*^ rats showed repetitive rotation (Fig. 1G) and reduced anxiety-like behaviors (Fig. 1I–K), which can be distinguished from deficit-SCZ [36, 37]. Moreover, our ASD model exhibited ASD phenotypes at early stages (4 weeks in age), and the diagnostic age in ASD patients is in general much earlier than that for SCZ patients [38].

Another important finding in this study is that the DAergic neurons were decreased in the midbrain of *Wnt1*^*sp/sp*^ rats. This decrease was detectable as early as embryonic day 12.5 (E12.5), and still existed at least at P90. Interestingly, the developmental loss of DAergic neurons in the midbrain was also observed in *Wnt1* KO mice, *Wnt1* naturally mutant mice (Sway mice), and *Wnt1* conditional KO mice [24, 39–42]. Moreover, the angle between the ventral midbrain (VM) and ventricular zone (VZ) was reduced in our *Wnt1*^*sp/sp*^ rats (Fig. S14), which, together with the loss of DAergic neurons, were both very similar to those observed in Lrp6 KO mice [43]. Lrp6 is one of the WNT ligand receptors. Similarly, knockout or inactivation of ß-catenin, or its direct downstream molecules, such as Otx2 and Lmx1a, all led to a significant loss of DAergic neurons in the midbrain [44, 45]. All those previous reports, together with our results, strongly suggested that molecules within the WNT signaling cascades, may constitute to a genetic or molecular basis for ASD. Accordingly, we suggest that a genetic detection of ASD risk or causative genes should be expanded to molecules that are implicated in both the WNT signaling pathway and the development of DAergic neurons.

The most important finding in this study came from our scRNA-seq. Although the role of WNT signaling in the midbrain and hindbrain patterning was already established [46], our results for the first time demonstrated that mutant WNT1 shifts the patterning of progenitor cells between the MHFP and ZHFP, and in turn, this patterning change may directly determine the development of DAergic neurons in the midbrain. These results are supported by another study, in which an activation of ß-catenin led to the expansion of the MHFP to the rostral region, although that study didn’t reveal whether the size of the hypothalamus is changed accordingly [47].

The mechanism for this patterning abnormality is still not clear. It should be noted that, however, in our study, we found that Dlk1, a non-classical ligand and inhibitor of Notch signaling pathway, was most significantly upregulated (Fig. 3E, Fig. S10B, G and H). It is well known that the Notch signaling plays an essential role in alternative cell fate determination between the neighboring cells [48]. Given that both the MHFP and ZHFP are neighboring one another, and that there are robust intersections between the WNT/ß-catenin and Notch signaling pathways [49], for instance the expression of Dlk1 was demonstrated to be inhibited by ß-catenin [50]. We have reasons to speculate that WNT1/ß-catenin inactivation resulted in the increased level of Dlk1 expression in one of the FP progenitor cells, while caused decreased expression of midbrain related genes, such as Foxa1, En1, Otx2, Lmx1a and etc. in the neighboring FP progenitor cells. At the same time, the Dlk1 highly expressed cells inhibit the neighboring cells specializing into midbrain floor plate cells. As a result, the progenitor cells in the MHFP should be decreased, which directly leads to the developmental loss of DAergic neurons in the midbrain and the Dlk1^+^ ZHFP progenitor cells increased. The finding that knockout of Dlk1 results in an ectopic location of DAergic neurons in the hypothalamus also indirectly supports our speculation [51]. The whole underlying pathogenic mechanism was illustrated in Fig. S15.

The most interesting finding is the demonstration of that DA-RT is effective for most, but not all, ASD-like behaviors. This is important, since it further proved that the reduction of the DA level caused by DAergic neuronal loss is the primary driver for the ASD-like behaviors. Given that the degeneration of DAergic neurons in the midbrain is the main reason for the motor dysfunction observed in Parkinson’s disease, and DA-RT is an effective strategy for these motor dysfunctions, it is possible that our effect might be directly related to the changes of the motor function. In our study, however, the locomotor activity was not changed after the DA-RT (Fig. 5E, Fig. S11B, C), indicating that the relieved ASD-liked behaviors were not due to the increased motor activity. The reason for this non-effect on motor function might be due to the short-term treatment. Recently, accumulating evidence has suggested that the DAergic system may be an important player in ASD, in both pathogenesis and treatment [52]. For example, the neural circuits involving DAergic neurons in the VTA region play a fundamental role in social behavior [53]. Consistently to our results, a significant decrease in the Th positive DAergic neurons, and an effective treatment with DA on the impaired social behavior was observed in an ASD model [54]. Moreover, these results are of a translational significance. Indeed, treatments with levodopa has been shown to be an effective choice for ASD patients, despite that this effect was only observed in a small proportion of patients [55]. However, we did not find an observable effect on repetitive and stereotyped rotation behaviors (Fig. 5H). The reason for this ineffectiveness is still unclear. One possibility is that an abnormal WNT1 function may also lead to a developmental defect in other types of neurons, such as neurons in the hypothalamus and cerebellum [40], rather than DAergic system only.

In summary, our results have provided new insights into the genetic, molecular, and neuronal mechanisms underlying ASD, as well as shed light on a new translational effort on the development of a new treatment for certain portion of ASD patients.

### Supplementary information

Supplemental Information
