# Supplementary material for: A novel mutation in intron 1 of Wnt1 causes developmental loss of dopaminergic neurons in midbrain and ASD-like behaviors in rats
Source: Mol Psychiatry. 2023 Sep 1;28(9):3795–805. doi: 10.1038/s41380-023-02223-8 (PMC10730402; doi:10.1038/s41380-023-02223-8)
Supplement: Supplementary file 1 — Supplemental Information [file 41380_2023_2223_MOESM1_ESM.pdf]

Supplementary Information

Title: A novel mutation in intron 1 of *Wnt1* causes developmental loss of dopaminergic neurons in midbrain and ASD-like behaviors in rats

Authors: Yongyi Li, Ph.D., [#,1,2,3], Mingwei Zhu, Ph.D., [#,3], Wenxiong Chen, Ph.D., MD., [#,4], Jing Luo, MS, [5], Xin Li, MS, [3,5,6], Yangyang Cao, MS, [7], Meng Zheng, MS, [3], Shanshan Ma, Ph.D., [1,2], Zhilan Xiao, MS, [3], Yani Zhang, MD., [4], Linyan Jiang, MS, [3], Xiumin Wang, Ph.D., [3], Ting Tan, Ph.D., [3], Xia Li, MS, [3],Qian Gong, BS, [3], Xiaoli Xiong, MD., [3], Jun Wang, Ph.D., MD., [7], Mingxi Tang, Ph.D., MD., [6,*], Mingtao Li, Ph.D., [1,2,*], Ya-Ping Tang, Ph.D., MD., [1,3,7,*]

Email: mxtang69@163.com; limt@mail.sysu.edu.cn;yptang12@gzhmu.edu.cn

Summary

The supplementary information file includes sections of Supplementary Methods, Supplementary Figures, Supplementary Tables and References.

Supplementary Methods

Human blood samples

All human subjects including ASD patients and their parents were recruited by the Department of Neurology in Guangzhou Women and Children's Medical Center (GWCMC), and the recruitment was pre-approved by the Committee of Medical Ethics in GWCMC. Clinical diagnostic algorithms or assessment of ASD was conducted based on the standards described in

DSM-V. All the peripheral blood samples were kept in deep freezing (liquid nitrogen) up to the uses.

Whole exome sequencing (WES) and sanger sequencing

The genomic DNA was extracted from the peripheral blood of ASD children and their parents using a genomic DNA extraction kit (Solarbio, D1700). WES of the proband's sample was performed at Beijing Genomics Institute (Shenzhen, China) as mentioned before in our published article (1). The primers used for the sanger sequencing were as follows: F: 5'-TCT TCT CAC TGC AGT CAG CG-3'; and R: 5'-GAG CTT CTG TCT TTG GGC TAG-3'.

Mutant gene identification

A total of 110 ASD patients examined in this study were children diagnosed with ASD according to DSM-5. Among those patients, one female child who was diagnosed as ASD when she was 3-years old exhibited deficits in communications, a low social responsiveness, repeatedly slapping on the thighs, belly, and head, irritability, and sleep disturbance. In an attempt to dig out possible risk/causative genes, the genomic DNA from the whole blood samples from all these patients, together with their parents (trio), were subjected to WES. Among over 30 new mutations identified from this screening (data not shown), a homozygous mutation in the intron 1 of *Wnt1* was identified, and the mutation was further confirmed by Sanger sequencing. Her parents were both heterozygous mutation and were both asymptomatic.

Animals

All the experiments on animals were conducted in accordance with the provisions for animal care and use described in "Guidance for the Care and Use of Laboratory Animal" issued by

NSFc, and were pre-approved by the IACUC in GWCMC (Ethics No.2019-23001). Humanized *Wnt1 c.104+1 G>A* mutant rats (Sprague-Dawley, SD) were generated by using the CRISPR/Cas9 Technology (Cyagen Biosciences Inc.). The rat *Wnt1* gene sequence was downloaded from Ensemble (ENSRNOG00000061818). The exon1 was selected as target site. Guide RNA (gRNA) targeting vector with targeting sequence, flanked by 120 bp homologous sequence combined on both sides is designed. The c.104+1G>A (TGG>TGA) in donor oligo will be introduced into exon1 by homology-directed repair. The mixture of gRNA, Cas9 mRNA generated by in vitro transcription and donor oligo were microinjected into fertilized eggs isolated from SD rats. The pups then were genotyped by PCR followed by sequence analysis. A female founder (F0) was generated, and then backcrossed to SD rats for at least three generations to avoid potential off-target mutations. The sequences used for CRISPR/Cas9 editing and the primers used for genotyping of the mutant site are listed in Supplementary Table1. All rats were raised in an SPF animal facility at GWCMC under the standard conditions:12 h of light/dark cycle, the temperature of 20-22 °C, the humidity of 60%, food and water ad libitum. Both male and female mutant rats, together with their littermate controls, were used throughout the experiments. The sample sizes of animals were determined based on the rules of 3R (reducing, reusing, recycling), and an adequate statistical power was guaranteed at the same time. Usually, tissue samples for molecular biological experiment were at least 3 in each group, animals for behavioral tests were at least 10 in each group, except in a few cases that the number of animals was below 10.

In vitro assay

EGFP genetic sequence, with Kpn1 and Bgl2 restriction enzyme cutting site in N and C terminal respectively, was amplified. The above EGFP sequence and pBudCE4.1 plasmid were double

digested by Kpn1 and Bgl2 respectively and then ligated, thus pBudCE4.1-EGFP plasmid was generated. The human genomic DNAs were extracted from the peripheral blood samples from a healthy people and the ASD patient who harbors the *Wnt1*$^{sp/sp}$ mutation. *Wnt1* WT and mutant genomic sequence was amplified, with ScaI and XbaI restriction enzyme cutting site in N and C terminal respectively. Then, the above *Wnt1*-nor and *Wnt1*-mutant PCR fragments were inserted into the intermediate vector pEGM-Teasy, name pEGM-Teasy-*Wnt1*-WT and pEGM-Teasy-*Wnt1*-mutant. The pBudCE4.1-EGFP, pEGM-Teasy-*Wnt1*-WT and pEGM-Teasy-*Wnt1*-mutant plasmids were double digested by ScaI and XbaI respectively. The enzyme-digested products of *Wnt1*-nor-myc and *Wnt1*-mutant-myc were separately cloned into pBudCE4.1-EGFP plasmids, and these plasmids were respectively named as *Wnt1*-normal-myc plasmid and *Wnt1*^{sp} -myc plasmid. All the elements of the plasmids were confirmed by sequencing. The pBudCE4.1-EGFP plasmid was used as the control. PCR primers used for these plasmids above are listed in Supplementary Table 2. The same amount in each plasmid above was transfected into 293T cells (purchased from and authenticated by Procell) using the lipo3000 system (Thermo Fisher Scientific). Transfected cells were harvested after culturing in DMEM (Procell) plus 10% FBS (Gibco) for 48 hours.

Rapid amplification of cDNA ends (5 'RACE)

A Roche 5'/3 'RACE kit (2nd, Cat. No. 03353621001) was used for 5 'RACE following the manufacturer’s instructions. Briefly, the total RNA of cells or tissues were extracted by Trizol and chloroform. RNA was precipitated by isopropanol and then dissolved in DEPC-treated distilled water. A human/rat *Wnt1*-specific primer 1 was used for a reverse transcription and the synthesized cDNA was purified by using a commercial kit (GenStar D206-01). A poly-A tail signaling was added to the 5’ end of the cDNA, and then amplified by PCR with a human/rat

Wnt1-specific primer 2 coupled with an oligo dT-anchor. A human/rat *Wnt1*-specific primer 3 and internal primers PCR-anchor were used for nested PCR. The PCR products were subjected to agarose electrophoresis and sequencing analysis. The primer sequences were listed in Supplementary Table 3. The *Wnt1* DNA and cDNA sequence were obtained from the PubMed (Human *Wnt1* cDNA sequence: NM_005430.4; Rat *Wnt1* cDNA sequence: NM_001105714.1; Human *Wnt1* genomic sequence: NG_033141.1; Rat *Wnt1* genomic sequence: NC_051342.1). Sequence alignment was done on DNAMAN (Version 9).

Real-time quantitative PCR (RT-qPCR)

The procedures for the total RNA extraction were described in 5'RACE. After removal of the genomic DNA, reverse transcription (RT) was conducted following the instructions of a RT kit (TaKaRa RR047A-1). The qPCR was performed according to the instructions of a qPCR kit (CWBIO CW3008S). Primer sequences are listed in Supplementary Table 4.

Chromatin co-immunoprecipitation (ChIP)

The experimental procedures were conducted according to the instruction of Magna ChIP G Tissue Kit (Millipore 17- 10085). Briefly, 3-4 brain tissues except forebrain from E12.5 embryos were dissected and pooled together. Tissue stabilizing solution and 1% polyformaldehyde were used for protein-DNA fixation and crosslinking. Then tissue was homogenized in tissue lysis buffer and remove the supernatant. The precipitate was resuspended in CHIP dilution buffer and the genomic DNA was fragmented via sonication. The sample above incubated with the mixture of protein G magnetic beads and anti-ß-catenin antibody in 4°C overnight with rotation. The above mixture was washed with low salt, high salt, LiCl, TE wash buffer in succession. Chromatin complex was eluted from the protein G magnetic beads with CHIP elution buffer plus

proteinase K. The de-crosslinked DNA was purified following the instruction of the kit (GenStar D206-01). The purified DNA was amplified by PCR with the primers followed, Otx2-F: 5'-TGT TCA AAG GCT TCG CTG GG-3'; Otx2-R: 5'-ACA CAC ACA CAC ACA CAA AAC TTC AG-3'. The PCR products were then subjected to agarose gel electrophoresis.

Western Blotting

Western blot was used to determine the expression level and the procedures were the same as our previous publication (2). Briefly, the targeted brain tissues were collected and homogenized in RIPA lysis buffer (Beyotime) with protease inhibitors. A total of 20 μg of protein from each sample was separated on 10% SDS-PAGE and transferred onto the PVDF membranes (Immobilon-P membranes, Millipore, Bedford). The membranes were blocked with 5% skim milk dissolved in PBS with 0.1% tween-20 for 1 hour at room temperature (RT), and then were incubated with a primary antibody overnight at 4°C, followed by an HRP-linked secondary antibody for 1 hour at RT. The blotting signal was visualized with an ECL detection system (Immobilon Crescendo Western HRP Substrate). Re-probe to anti-GAPDH or ß-Tubulin antibody was used to normalize the protein-loading amount. Densitometry was performed with using Image-J (win 64) to determine the expression level. The primary antibodies included: β-catenin (CST8480) at 1:1000, Myc (CST2276) at 1:500, GFP (CST2956S) at 1:500, WNT1 (Proteintech 27935-1-AP) at 1:500, TH (Millimore AB152) at 1:1000, Dlk1 (Santa Cruze sc376755) at 1:500, Nkx2-1 (Abcam ab76013) at 1:1000, Foxa1 (Abcam 170933) at 1:1000, Otx2 (R&D System AF1979) at 1:500, β-Tubulin (CST86298S) at 1:5000, and GAPDH (CST174S) at 1:5000. Secondary antibodies included HRP-goat anti-rabbit IgG (Biodragon BF03008) at 1:5000, and HRP-goat anti-mouse IgG (Biodragon BF03009) at 1:5000.

Chemical administration

Levodopa (Topscience T0848) and Carbidopa (Topscience T6795) powder were dissolved in PBS containing 10mg/ml Vitamin C (Sigma) to delay the oxidation reaction. Carbidopa (12.5 mg/kg) was administered (i.p.) 30 minutes before levodopa (50 mg/kg) was given (i.p.)(3). Both compounds were used once a day, for 3 consecutive days. The behavioral test was conducted 2 hours after the last injection of levodopa. For USV test, because the most obviously behavioral phenotype was observed in P11 (Fig. 1A), here we chose this time point to examine the efficacy of the DA-RT. Pups at P8 were subjected to the treatment, and behavioral test were performed at P11.

DA and its metabolites

DA and its metabolites were detected by using HPLC-MS, and the procedures were described previously. Briefly, a total of 10 mg of striatal tissue was homogenized in 20-fold volume of 2% formic acid solution on ice. A total of 10 μl of the mixture containing isoproterenol (concentration of 40 ng/ml), 10 μl 0.1% acetic acid, and 100 μl methanol was used as the internal standard. The tissue samples were centrifuged, and the supernatants were collected, and then subjected to HPLC-MS. The standard substances of DA, homoprotocatechuic acid (DOPAC) and homovanillic acid (HVA), were purchased from Sigma Aldrich. Data were collected by using Vanquish ultra-high performance liquid chromatography and TSQ Quantis triple quadrupole tandem mass spectrometer (ThermoFisher Company of the United States). The chromatographic column adopted X Select CSH C18 (100mm × 2.1mm, 2.5μm, Waters, the United States).

Immunofluorescent staining and Immunohistochemistry

The procedures for both immunofluorescent staining and immunohistochemistry were described previously (4). Briefly, rats were anesthetized with ketamine (100 mg/kg)/xylazine (20 mg/kg) mixture and were perfused transcardially with 0.9% saline, followed by 4% paraformaldehyde (PFA). Brains were post-fixed in 4% PFA overnight and then equilibrated with 30% sucrose in phosphate buffer overnight. Coronal brain sections (20 µm) of rats at P28, or 10 µm in thickness for rats at E12.5, were made with a Cryostat (Leica 3050S). For immunofluorescent staining, sections were permeabilized in PBS with 0.3% Triton X-100, and were blocked with 5% goat serum in PBS with 0.1% Triton X-100 for 1 hour. The sections were then incubated in PBS with a primary antibody overnight at 4°C, and followed by a fluorescent secondary antibody for 1 hour at room temperature. After washing, the slides were mounted with an anti-fade mounting medium (Electron Microscopy Sciences), and the fluorescent images were captured by a confocal microscopy (Leica SP8). The primary antibodies used included: TH (Millipore AB152) at 1:1000, Aldh1a1 (Proteintech 15910-1-AP) at 1:200, Dlk1 (Santa Cruze sc376755) at 1:200, and Foxa1 (Abcam 170933) at 1:400 fold-dilution. The secondary antibodies used were: Goat anti-rabbit IgG (Alexa Fluor 488; Abcam 150081) at 1:1000, Goat anti-mouse IgG (Alexa Fluor 555; Abcam 150114) at 1:1000 fold-dilution. For immunohistochemistry, the endogenous peroxidase was inactivated by using 3% hydrogen peroxide in PBS, and the sections were permeabilized in PBS with 0.3% Triton X-100, and blocked with 5% goat blocking serum in TBS with 0.1 Triton X-100 for 1 hour. After all these, the sections were incubated with primary antibody in PBS overnight at 4°C, followed by an HRP-linked rabbit/mouse secondary antibody (Dako REAL EnVision Detection System, k5007) for 1 hour at RT. Then, the Dako REAL Substrate Buffer containing hydrogen peroxide combined with DAB was used for chromogenic reaction. Finally, slides were dehydrated with alcohol gradient and mounted with neutral resin.

The primary antibodies were: TH (Millipore AB152) at 1:3000, Nkx2-1 (Abcam ab76013) at 1:500, Foxa1 (Abcam ab170933) at 1:500, and Otx2 (Proteintech 13497-1-AP) at1:500. Images were captured by an inverted microscope (Leica DMi8). All captured images were analysed with Image-J software (win 64).

Behavioral tests

All rats in each genotype were randomly numbered and grouped by experimenters who would not conduct the behavioral tests. Experimenters who conducted the behavioral tests were blind to the genotype of each animal until data analysis. In order to avoid any potential effect from the behavioral test, animals used for behavioral tests only for one test. Tests were conducted between 9:00-18:00 in a sound- and light-proved behavioral room.

1) Ultrasonic vocalization (USV) test. A protocol of isolation-induced USV in pups was used, and the procedures were described in our previous publication (5). Briefly, neonates were examined following a brief maternal separation on P2, P5, P8, P11, and P14. USVs from individually isolated pups were recorded using an externally polarized condenser microphone with a frequency range of 30 to 300 kHz that was attached 15 to 20 cm above the floor of a housed chamber. The output information from the microphone was transferred into an Avisoft-Ultrasound Gate recording system (Avisoft Bioacoustics), and the pup-emitted calls were recorded to WAV sound files using parameters optimized for rats. Pups were individually placed in the sound-proof chambers, and calls were recorded for 300 seconds. Data were analyzed using a generalized linear model with a negative binomial distribution and a log-link function. Data were exported and processed by SAS Lab Pro (Version 5.2.10; Avisoft Bioacoustics, Germany).

2) Three-chamber test. The procedures for this test were described in our previous publication (5). Briefly, test rats at the age of 4 weeks were used to assess sociability and preference for social novelty. Demo subjects (stranger 1 and stranger 2), at the same age, were first habituated in a cylindrical cage of the device for three days, where the stranger 1 and stranger 2 would be place there during the data collecting stage. Test rats were placed in the testing room for 1 day prior to the data collecting stage. This stage began with a 10-min freely-moving phase in three chambers of the device, in order to habituate to the environment. For the sociability test, a demo rat (stranger 1) was randomly put into one of the two wire cages, and then a test rat was introduced to the middle chamber, and allowed to freely explore the environment for 10 min. Following this, another demo rat (stranger 2) was introduced into the other wire cage, and the test animal was allowed to freely explore for another period of 10 min. Parameters including the time spent in each chamber was recorded. All these parameters, together with the track maps were analyzed by using an automated SMART software.

3) Open-field test. The procedures for this test were described in our previous publication (6). Briefly, an automatic-recording open-field working station (MED Associates) was used. The open-field box (50×50×30 cm high) was divided into 16 identical squares by invisible but computer-detectable lines, and the open-field was illuminated by a dim light (20 lux). The central 9000 cm^3 (30 cm * 30 cm * 30 cm) was defined as the central area. Two sets of 16 pulse-modulated infrared photobeam were placed on opposite walls 2.5 cm apart from the wall to record X-Y ambulatory movements. Exploratory behavior in the box was computer-interfaced at a sampling rate of 100-ms resolution. Rats at 6 weeks in age were transported to the behavioral room to adapt the environment for at least 1 hour before the experiment. Behavioral indices including total distance traveled, ambulation counts, and number of rearing were recorded

automatically by the scanning system for 30 minutes. Panlab Smart 3.0 software recorded the distance, speed, and time in the central and peripheral area. Numbers of rotation, grooming were manually counted based on videos.

Single-cell RNA sequencing (scRNA-seq)

We conducted scRNA-seq in Guangzhou Yuanxin Biotechnology Company, China. The steps are briefly described as follows:

1) **Dissection of Embryonic 11.5 (E11.5) brains.** Following a timed mating, each pregnant dam at E11.5 was anesthetized with 10% chloral hydrate, and the entire uterus was dissected out and immediately placed into ice-cold PBS. Individual embryos were carefully collected, and were placed into ice-cold PBS. Brain tissues including the hypothalamus, midbrain, and hindbrain but not the forebrain, were dissected under a stereomicroscope. Tissues were quickly stored in liquid nitrogen, and single-cell isolation was performed within 24 hrs.

2) **Nuclear extraction.** The procedures for nuclear extraction were followed up by the manufacturer's instruction. Briefly, tissue was homogenized in Hibernate E containing B27 and GlutaMAX by using a Pasteur pipette on ice, and cell debris were first removed by filtering with a 30 μm MACS SmartStrainer. The suspension was then centrifuged at 500 rcf for 5 min, 4 ºC. After discarding the supernatant, the nuclei were washed and resuspended with a resuspension buffer gently. Then, Myelin was removed using Myelin Removal Beads II and a single LS column. Sucrose Cushion Buffer I was added to the nuclei followed by density gradient centrifugation, so that the final density is 700-1200 nuclei/μl.

3) **Preparation of cDNA libraries and sequencing.** The nuclei extracted above were processed

following the instructions of Chromium Next GEM Single Cell 3′ Reagent Kits v3.1 (10× GENOMICS) to generate cDNA libraries. Briefly, each nuclear suspension was adjusted to 1000 nuclei/μl, and was loaded onto the Chromium Controller instrument to generate single-cell gel bead in emulsions (GEMs), and individual nuclear was then separated into droplets along with gel beads coated with cell barcode tags (10× cell barcode), Unique Molecular Identification Tag (UMI) and poly (dT) sequences. GEM reverse transcription was performed on a Veriti 96-well thermal cycler (Thermo Fisher Scientific, Waltham, MA, USA). The cDNA library was then amplified using primers for the R1 and P5 arms after a reverse transcription. The cDNA library was fragmented, end repaired, and poly A-tailed. Adapters were then ligated after selecting fragments of appropriate length. Sample index PCR was performed and the SPRI selection beads were used for a final purification. Finally, the sequencing library was sequenced by using an Illumina NovaSeq 6000 platform.

4) Read alignment and quality control. Raw reads of each sample were aligned to the rat genome (Rnor_6.0), and the gene expression matrices were generated for each sample by the cellranger (v3.1.0) count function with default parameters. To determine the fraction of ambient RNA in each single cell, the SoupX package (v1.5.2) was used with default parameters. The RNA expression in each cell was then corrected using the ambient mRNA expression profile and estimated contamination. After this correction, we used DoubletFinder (v2.0.3) to identify doublet cells. After removing candidate doublets, we discarded cells with low quality, based on the criteria: unique molecular identifiers (UMI) lower than 800 or upper than 4000. To exclude ribosomal genes from contributing to the clustering, we removed ribosomal genes from the expression matrix.

5) Cell clustering and cell-type annotation. The R package Seurat (v3.0.2) was used to cluster the cells in the merged matrix of data. From the filtered cells, the gene expression matrixes were

normalized to the total UMI counts per cell and transformed to the natural log scale. To correct the batch effects, we integrated different samples using reciprocal PCA (rPCA) implemented in Seurat. We used the FindVariableFeatures function to obtain the top 2000 highly variable genes (HVGs) of each sample, and as the input dataset for batch effect correction. Using the FindIntegrationAnchors function the default dimensions (1:20), we found a set of pairwise correspondences between individual cells. These anchors are used for downstream integration of the objects. We used the IntegrateData function with the previously computed anchor set as a parameter to integrate all sample Seurat object. The default dimensions parameters (1:20) was used for the anchor-weighting procedure. The integrated dataset on all cells were then used to scale and center the genes, compute the principal components (PCs). After PCA to reduce dimensionality and build k-nearest neighbor graphs (k =20) of the cells with the function FindNeighbors based on the Euclidean distance in the 50-dimensional PC space, the main cell cluster was identified using the Louvain-Jaccard graph-based method. For classifying all filtered cells, we set the clustering parameter resolution to 0.3 with the function FindClusters in Seurat. Next, the function RunUMAP with dimensions parameters (1:20) in Seurat was used to reduce high-dimension into two-dimension (2D) for visualization. Lastly, we run the Seurat FindAllMarkers function with the default parameters to identify the genes specifically expressed in each cluster. The significance of the differences in gene expression was determined using the Wilcoxon rank sum test with Bonferroni correction, and cell types were manually annotated based on the cluster markers. A marker-cluster heatmap was generated with the R pheatmap (v1.0.12) package. To calculate the sample composition based on cell type, the number of cells for each cell type from each sample were counted. The counts were then divided by the total number of cells for each sample and scaled to 100% for each cell type.

6) Analyses of differential expression of genes (DEGs). DEGs of two groups with each cell type were performed using the FindMarkers function in Seurat. The significance of the differences in gene expression was determined by using the Wilcoxon rank sum test with Bonferroni correction. The different genes of two groups in each subcluster were determined based on following criteria: 1) expressed in more than 10% of the cells within either or both two groups; 2) $|\log2FC| > 0.25$; and 3) Wilcoxon rank sum test adjusted p-value < 0.05.

Statistical analysis

Before statistical analyses, all data were subjected to a Shapiro-Wilk test and F test to show distribution pattern, homogeneity of variance, as well as to determine statistical methods that will be used. A comparison between two groups was performed by using unpaired Student's *t*-test. Multiple comparisons were performed by using one-way ANOVA, followed by *post-hoc* Duncan's test. For the results of Western blot and RT-qPCR, we first adjusted the variation of signaling intensity from different batch of experiments, and the expression level in each experiment was then normalized by an average level from all WT samples. Statistical analyses were conducted by using the Graphpad Prism (Version 8.0). All data are presented as mean ± SEM, and $p < 0.05$ is considered as a significant difference.

Supplementary Figures

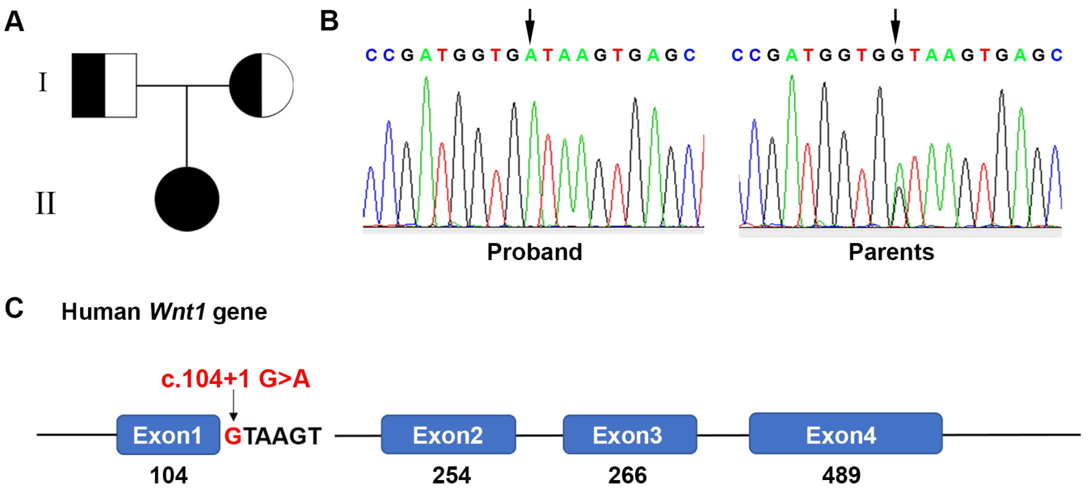

Fig. S1. A novel *Wnt1* intron mutation. A. Pedigree of ASD proband. B. Chromatograms of sanger sequencing of the proband and her parents, the black arrow points to the mutation site. C. Structure of human *Wnt1* gene. The blue rectangles represent the exons, the black line represents introns, and the red arrow marks the mutation site.

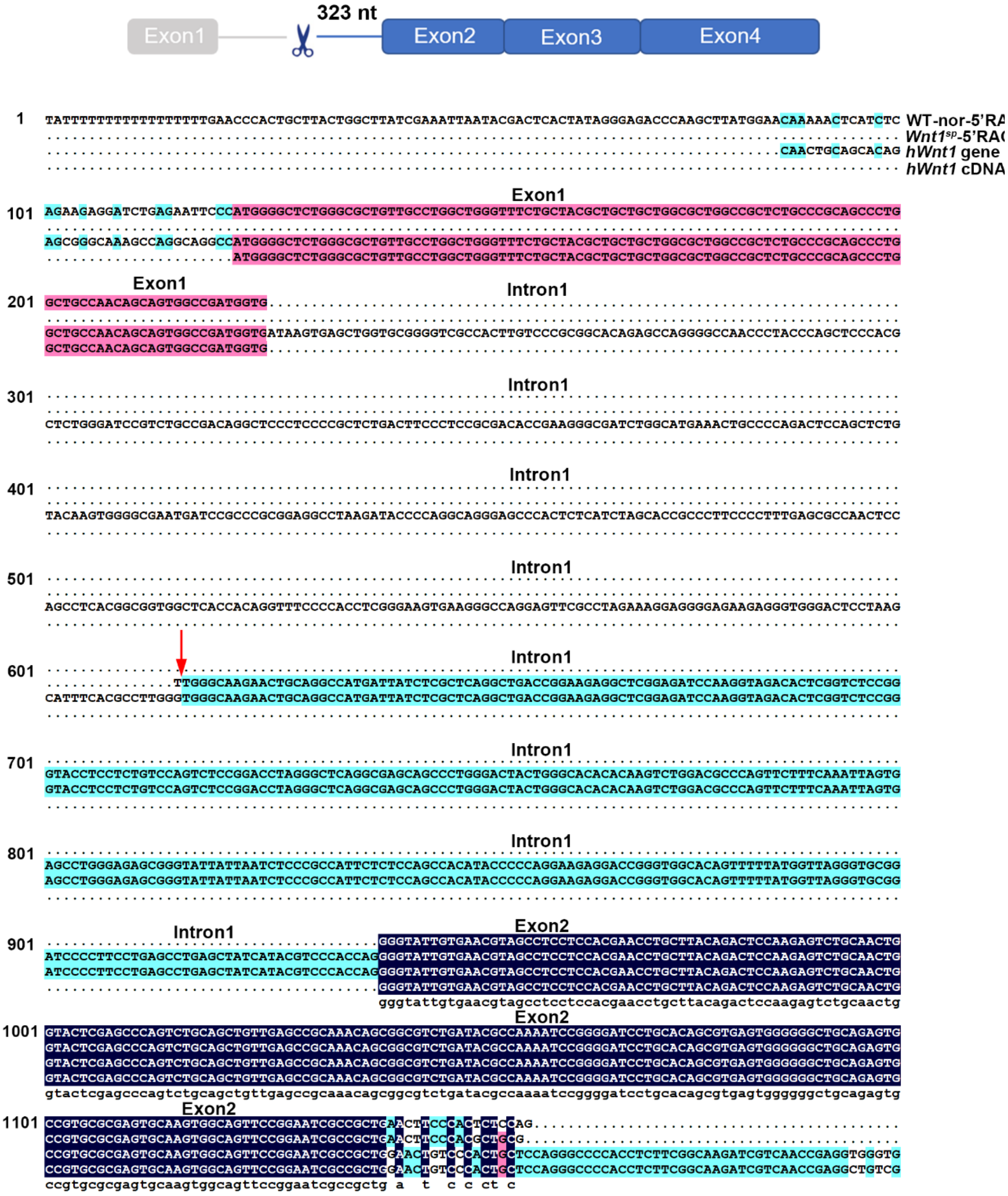

Fig. S2. Sequence alignment of *Wnt1*-nor and *Wnt1*^{sp} 5'RACE products. Upper panel is a schematic diagram of the mutated *Wnt1* mRNA. The lower panel is the sequence alignment

among *Wnt1*-nor 5’RACE products, *Wnt1*^{sp} 5’RACE products, *hWnt1* gene and *hWnt1* cDNA. The sequence in pink is exon 1, followed by intron 1, sequence in cyan is the retained intron 1, sequence in dark blue is exon 2, and red arrow indicates the the new splicing site.

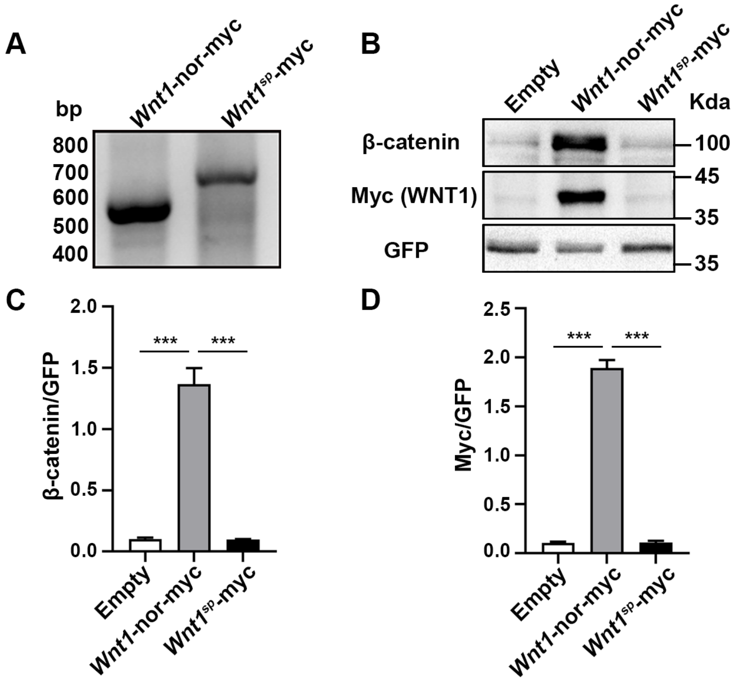

Fig. S3. The effect of the mutation on the expression of WNT1 in vitro. A. A 5'-RACE reveals different lengths of mRNA products in two sets of 293T cells that were respectively transinfected with *Wnt1*-nor-myc (n = 3) and *Wnt1^{sp}*-myc plasmids (n = 3). B. Western blot shows that although GFP expressed in both sets of 293T cells described above, the expression of either ß-catenin or Myc (WNT1) was at the marginal level in cells transinfected with the mutant one, in contrast to the robust level in the cells transinfected with the WT one. C and D. Quantitative analyses of the expression level of ß-catenin (n = 3) (C) and Myc (WNT1) (n = 3) (D).

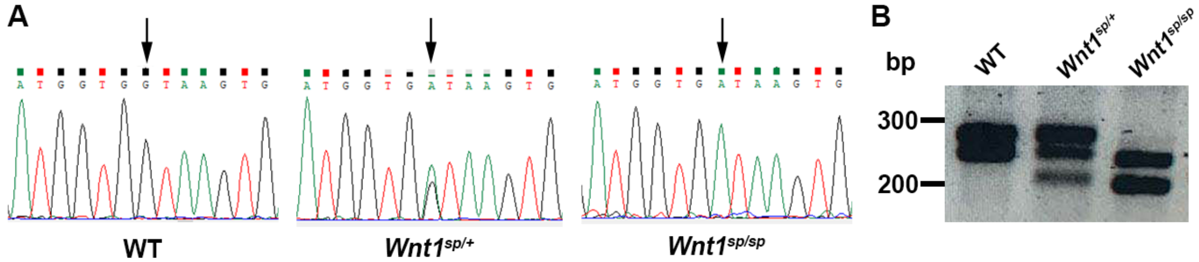

Fig. S4. Genotyping the rats. A. Sequencing the PCR products from the genomic DNA of WT, *Wnt1*$^{sp/+}$, and *Wnt1*$^{sp/sp}$ rats, the black arrow points to the mutation site. B. Agarose gel electrophoresis of PCR products digested with restriction enzyme (Hph1).

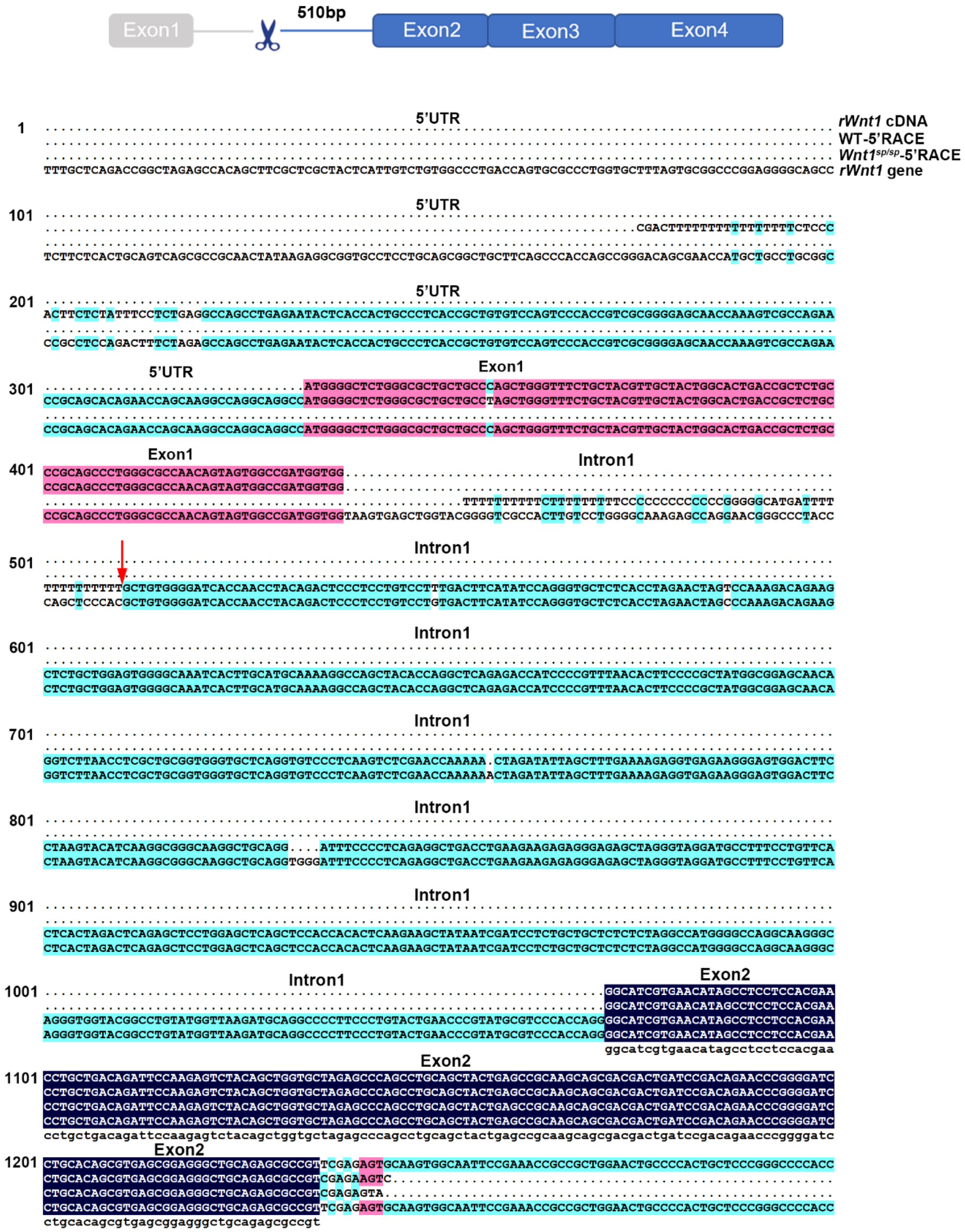

322 **Fig. S5. Sequence alignment of 5' RACE products of brain tissue in WT and *Wnt1*$^{sp/sp}$**

323 **rats.** The upper panel shows the structure of *Wnt1* mRNA in *Wnt1*$^{sp/sp}$ rats. The below panel is

the sequence alignment of rat *Wnt1* cDNA, rat *Wnt1*-WT 5’RACE products, rat $Wnt1^{sp/sp}$ 5’RACE products, rat *Wnt1* gene. The sequence in pink is exon 1, followed by intron 1, sequence in cyan is the retained intron 1, the sequence in dark blue is exon 2, and the red arrow points to the new splicing site (n = 3).

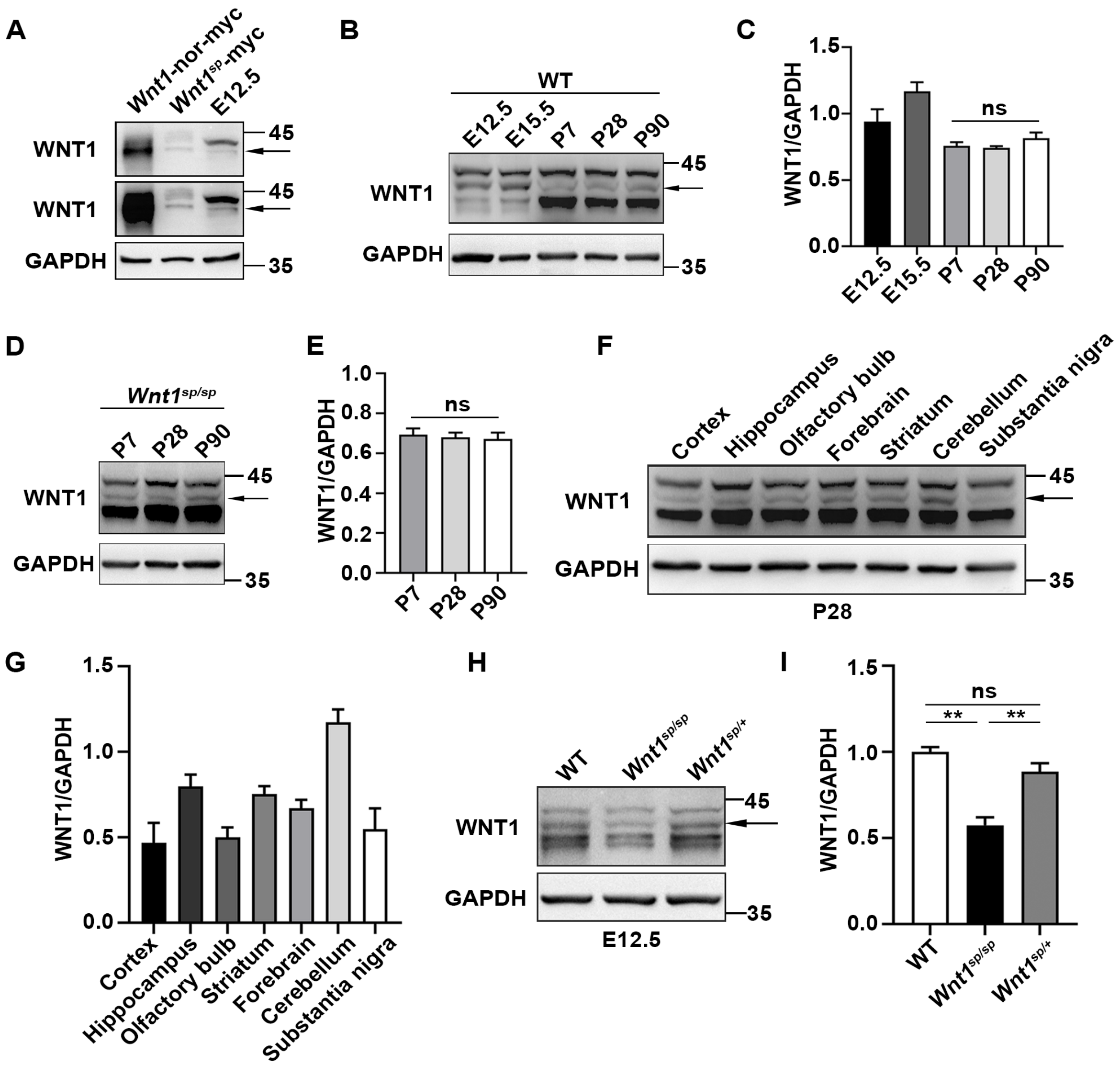

Fig. S6. WNT1 expression level in vivo. A. The specificity of WNT1 antibody was verified by overexpression of *Wnt1*-nor-myc and *Wnt1*^{sp}*-myc* plasmids in 293T cells. The short exposure image is on the upper panel and the long exposure image is on the middle panel. B. WNT1 expression level in WT rat brains at E12.5, E15.5, P7, P28, and P90. C. Quantitative analyses of B (n = 3-4 in each group). D. WNT1 expression level in *Wnt1*$^{sp/sp}$ rat brains at P7, P28, and P90. E. Quantitative analyses of D (n = 3 in each group). F. WNT1 expression level in different brain regions at P28. G. Quantitative analyses of F (n = 3 in each group). H. WNT1 expression level in WT (n = 3), *Wnt1*$^{sp/sp}$ (n = 3), and *Wnt1*$^{sp/+}$ (n = 4) brains at E12.5. I. Quantitative

analyses of H. All black arrow indicates the specific WNT1 signal. All data are expressed as mean ± SEM. **, $p < 0.01$; ***, $p < 0.001$. One way ANOVA followed by Duncan's test.

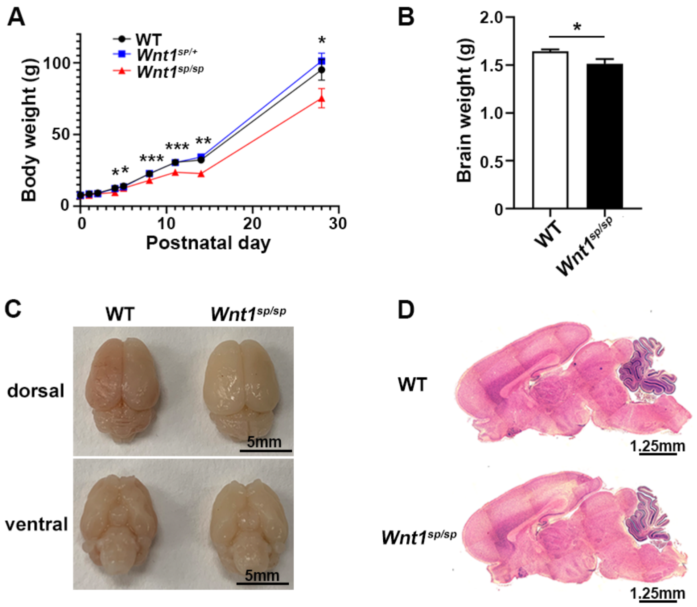

Fig. S7. Overall condition in *Wnt1*$^{sp/sp}$ rats. A. Body weight of WT (n = 8-20), *Wnt1*$^{sp/+}$(n = 10-20), *Wnt1*$^{sp/sp}$ (n = 5-20) rats at different postnatal days. B. Brain weight of WT (n= 10) and *Wnt1*$^{sp/sp}$ (n= 8) rats at P28. C. Dorsal and ventral view of the brain in WT (n = 4) and *Wnt1*$^{sp/sp}$ rats (n =3). D. H&E staining of sagittal section of brain in WT and *Wnt1*$^{sp/sp}$ rats (n = 3 in each group). Data are expressed as mean ± SEM, and *, $p < 0.05$; **, $p < 0.01$; ***, $p < 0.001$. Student's *t*-test or one way ANOVA, followed by *post-hoc* Duncan's test.

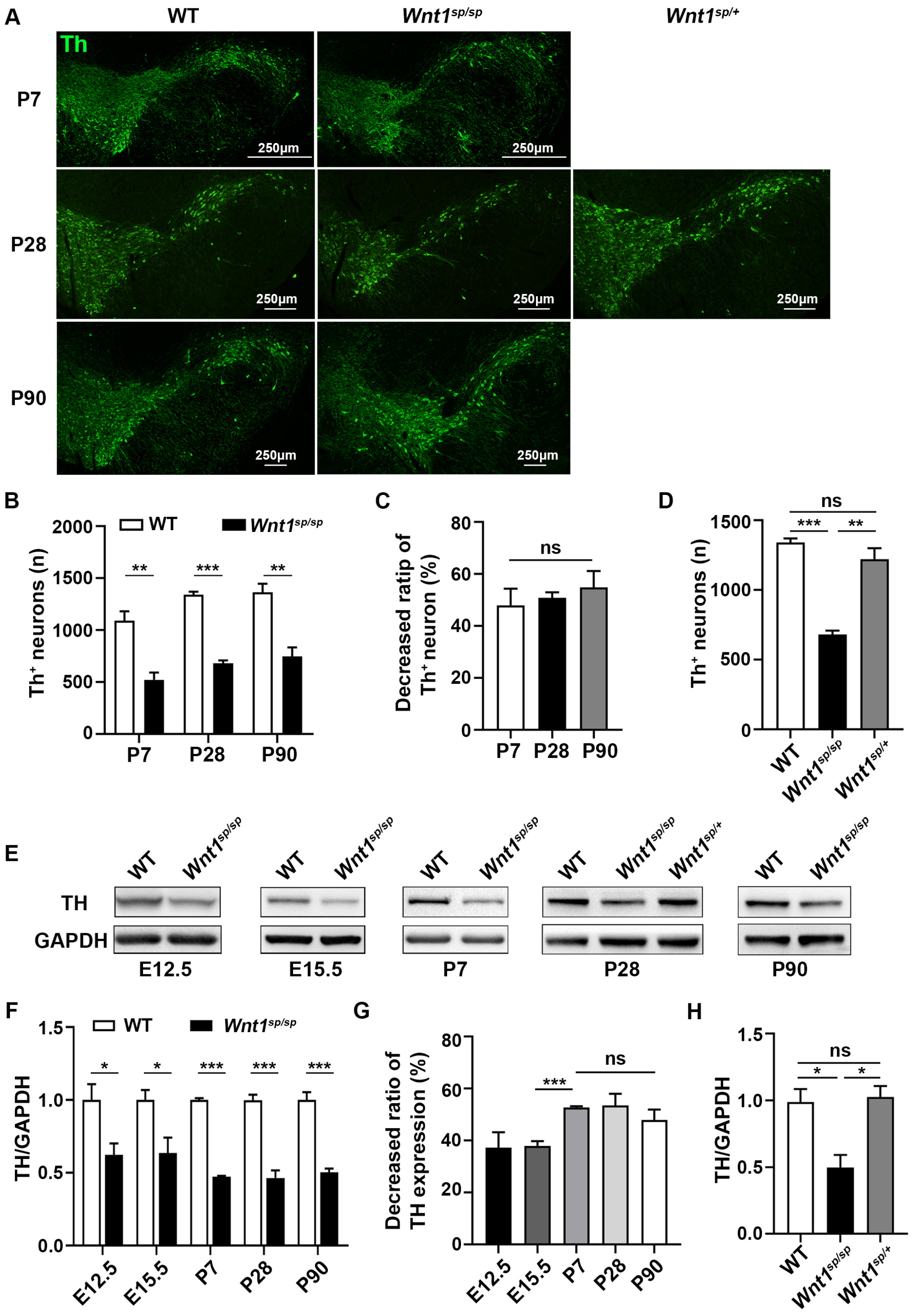

Fig. S8. The loss of Th$^+$ dopaminergic neurons in mutant rats was not further enhanced after the birth. A. Th immunofluorescence staining of brain sections in rats at P7 and P90 (WT:

n = 4, *Wnt1*$^{sp/sp}$: n = 4) or at P28 (WT: n = 4, *Wnt1*$^{sp/sp}$: n = 4, and *Wnt1*$^{sp/+}$: n = 5). Quantitative analyses of Th^{+} neurons (B) and changed ratio (C) in WT and *Wnt1*$^{sp/sp}$ rats at P7, P28, and P90. D. Quantitative analyses of Th^{+} neurons in WT, *Wnt1*$^{sp/sp}$, and *Wnt1*$^{sp/+}$ rats at P28. E. Western blot shows the expression of Th in the midbrain of rats at E12.5 (n = 3), E15.5 (n = 4), P7 (n = 4), P28 (n = 7), and P90 (n = 5). F. Quantitative analyses of Th expression level in WT and *Wnt1*$^{sp/sp}$ rats at E12.5, E15.5, P7, P28, and P90. G. The decreased ratio of Th expression in *Wnt1*$^{sp/sp}$ rats compared with WT rats at E12.5, E15.5, P7, P28, and P90. H. Quantitative analyses of Th expression in WT (n = 10), *Wnt1*$^{sp/sp}$ (n = 7) and *Wnt1*$^{sp/+}$ (n = 5) rats at P28. All data are expressed as mean ± SEM. *, $p < 0.05$; **, $p < 0.01$; ***, $p < 0.001$. Student's *t*-test or One way ANOVA followed by *post hoc* Duncan's test.

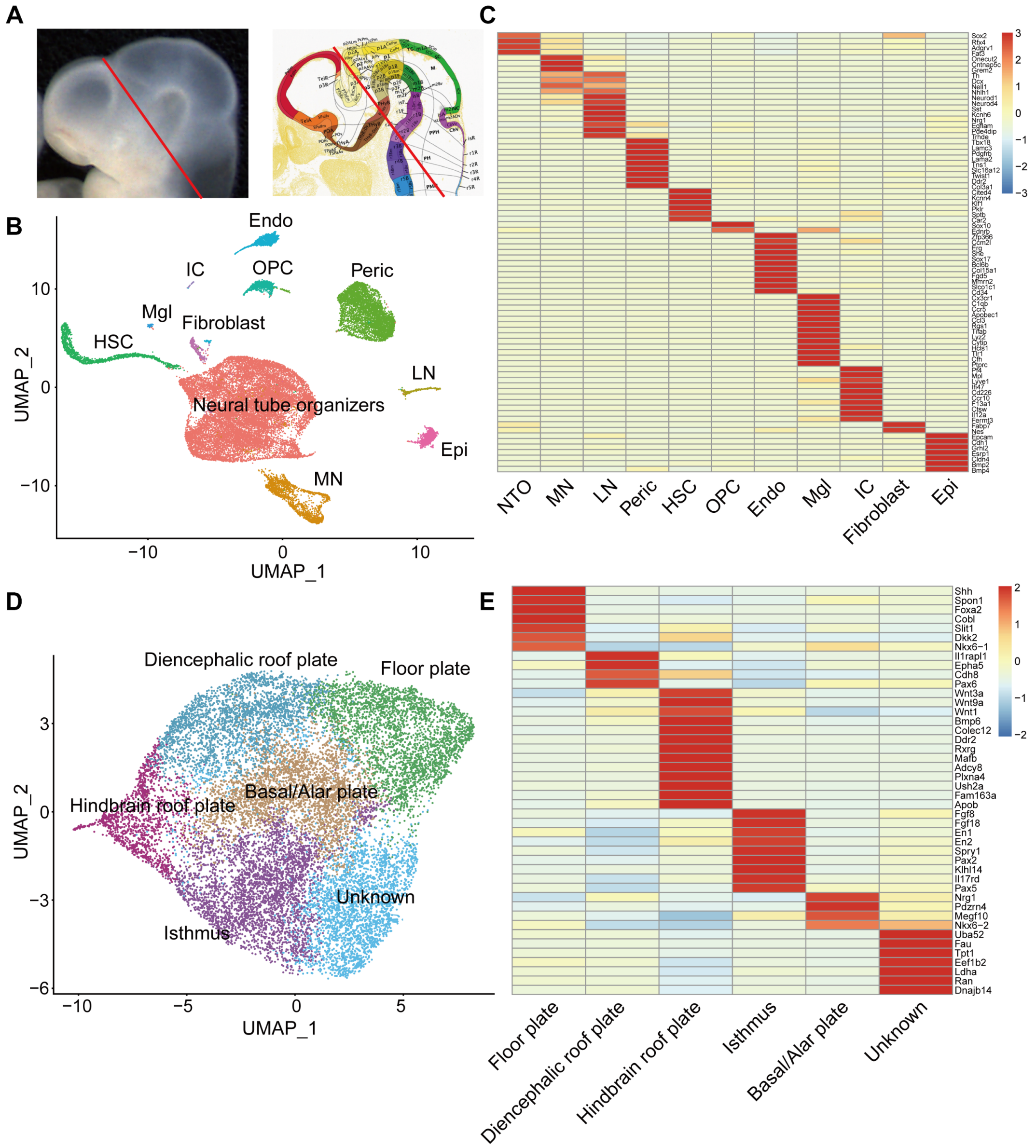

Fig. S9. Single-cell RNA sequencing (scRNA-seq) of progenitor cells in rats at E11.5. A. Poly-RNA from brain tissues including the diencephalon, midbrain, and hindbrain, but not the forebrain, at E11.5 were extracted and used for scRNA-seq; B. UMAP plot of all cells assayed, colored by annotated cell type identity. MN: Medial neuroblast; LN: Lateral neuroblast; Peric: Pericytes; HSC: Haematopoietic stem cells; OPC: Oligodendrocyte epithelial cell precusor cells; Endo: Endothelial cells; Mgl: Microglia; IC: Immune cells; Epi: Epithelial cells. C. Heatmap

shows the marker genes of each cell cluster. NTO: Neural tube organizer. D. UMAP plot of neural tube organizer cluster, colored by annotated cell type identity; E. Heatmap shows the marker genes of each subcluster.

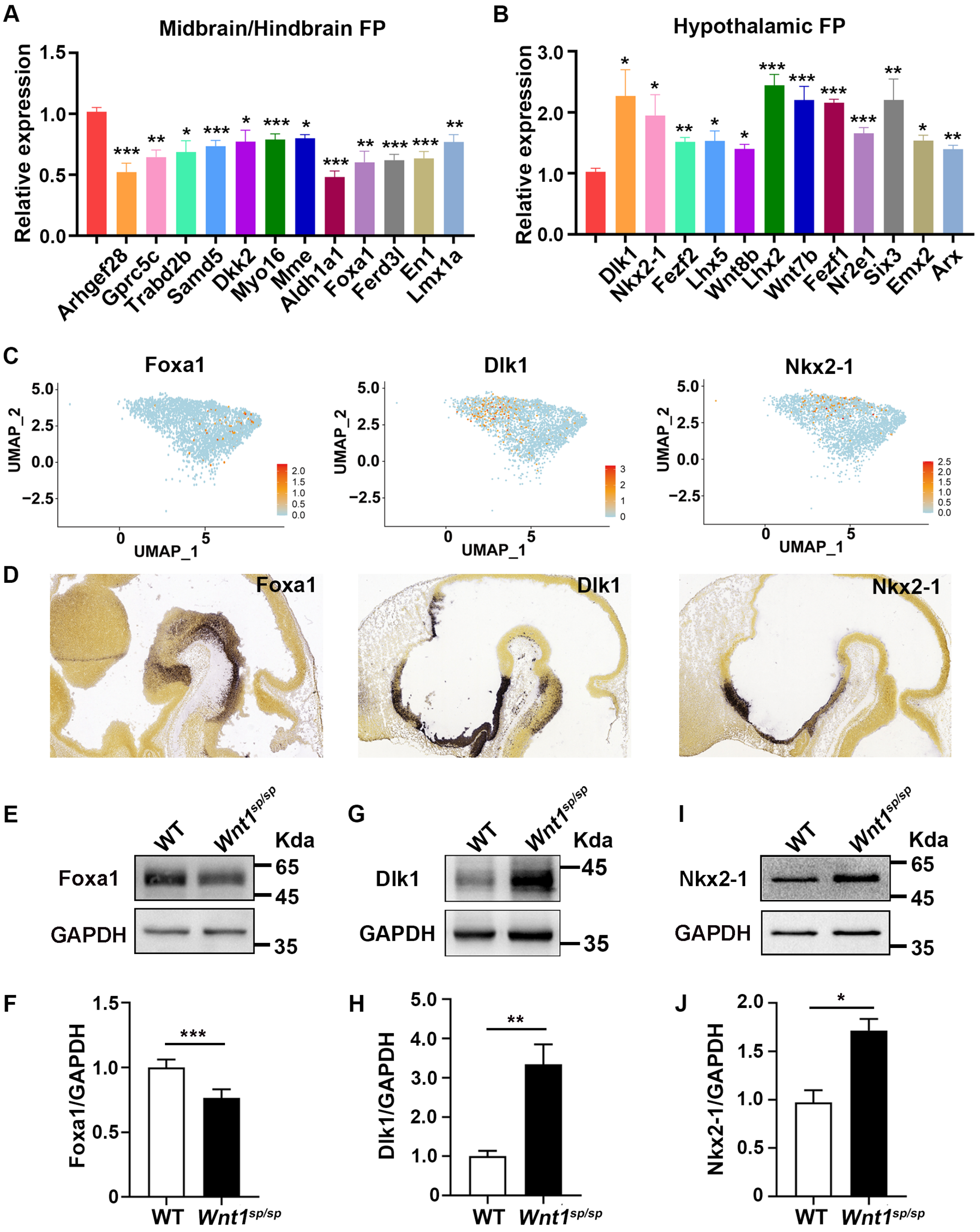

Fig. S10. A changed patterning of progenitor cells in the midbrain was observed in *Wnt1^sp/sp^* rats. E12.5 Embryonic brain tissue including hypothalamic, midbrain, and hindbrain (Figure S9A) were used for RT-qPCR and Western blot. A. The expression level of MHFP marker genes

(n = 3-6) detected by RT-qPCR in E12.5 embryonic brain tissue. B. The expression level of ZHFP marker genes (n= 3-6) detected by RT-qPCR in E12.5 embryonic brain tissue. The expression level of the corresponding gene in the WT brain was always set as 1. C. The feature plot of Foxa1, Dlk1 and Nkx2-1. D. In situ hybridization of Foxa1, Dlk1 and Nkx2-1 from Allen brain atlas-Developing Mouse Brain data portal. E-F. Western blot detected the expression level of Foxa1 (E) and the corresponding densitometric analysis (F) (n = 3). G-J. Western blot detects the expression level of Dlk1(G) and Nkx2-1 (I) and the corresponding densitometric analysis (H, J) (n = 3). Data are expressed as mean ± SEM. *, $p< 0.05$, **, $p < 0.01$, ***, $p<0.001$. All Student's *t*-test.

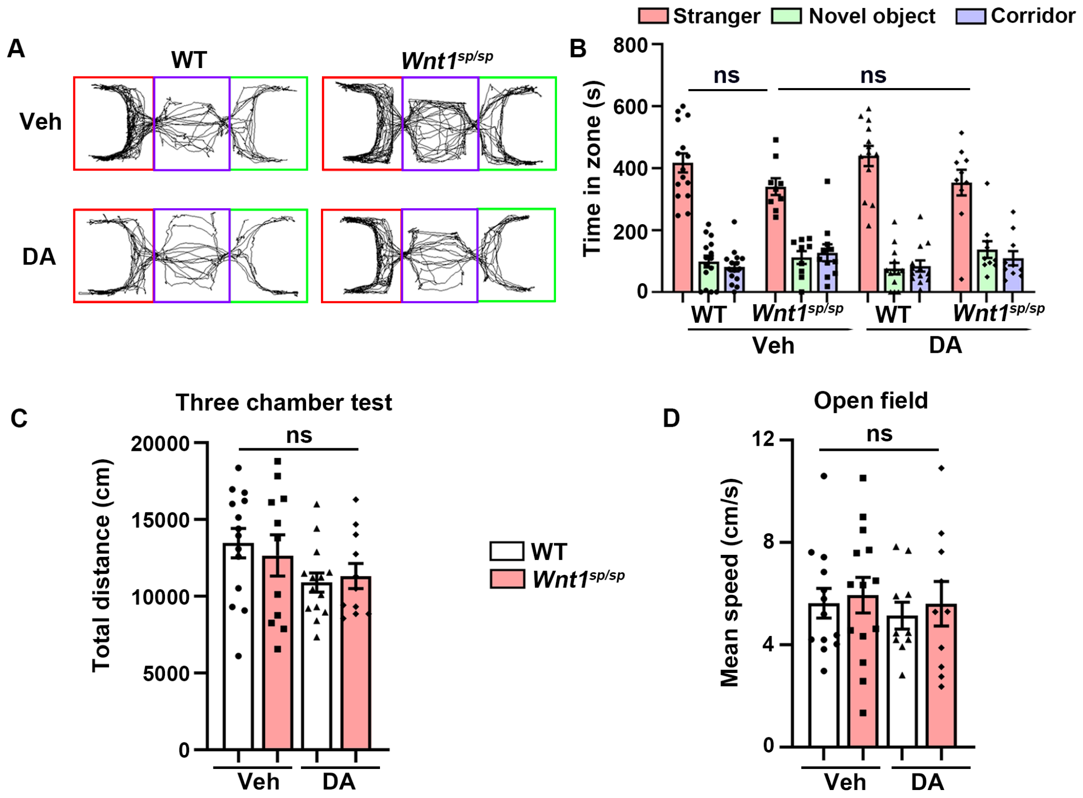

Fig. S11. Effect of DA-RT on three chamber social preference test and motor activity. A. Social preference test. The left panel shows the trajectory diagram of the test. A stranger was placed in the left chamber (red), and the right chamber (green) remained empty. A test rat was placed in the middle chamber (purple). The right panel shows time spent in each chamber. Both WT (n = 13-15 in a group) and *Wnt1*$^{sp/sp}$ rats (n = 9-10 in a group) were treated with vehicle (Veh) or DA-RT (DA), and then behavioral responses were recoded as described in Fig. 5A. B. Total distance rats traveled in three chamber, WT (n = 13-14 in a group) and *Wnt1*$^{sp/sp}$ rats (n = 10-11 in a group); C. Mean speed rats moved in the open field, WT (n = 10-12 in a group) and *Wnt1*$^{sp/sp}$ rats (n = 10-14 in a group). Data are expressed as mean ± SEM. *, p< 0.05, **, p < 0.01, ***, p<0.001. All Student's *t*-test.

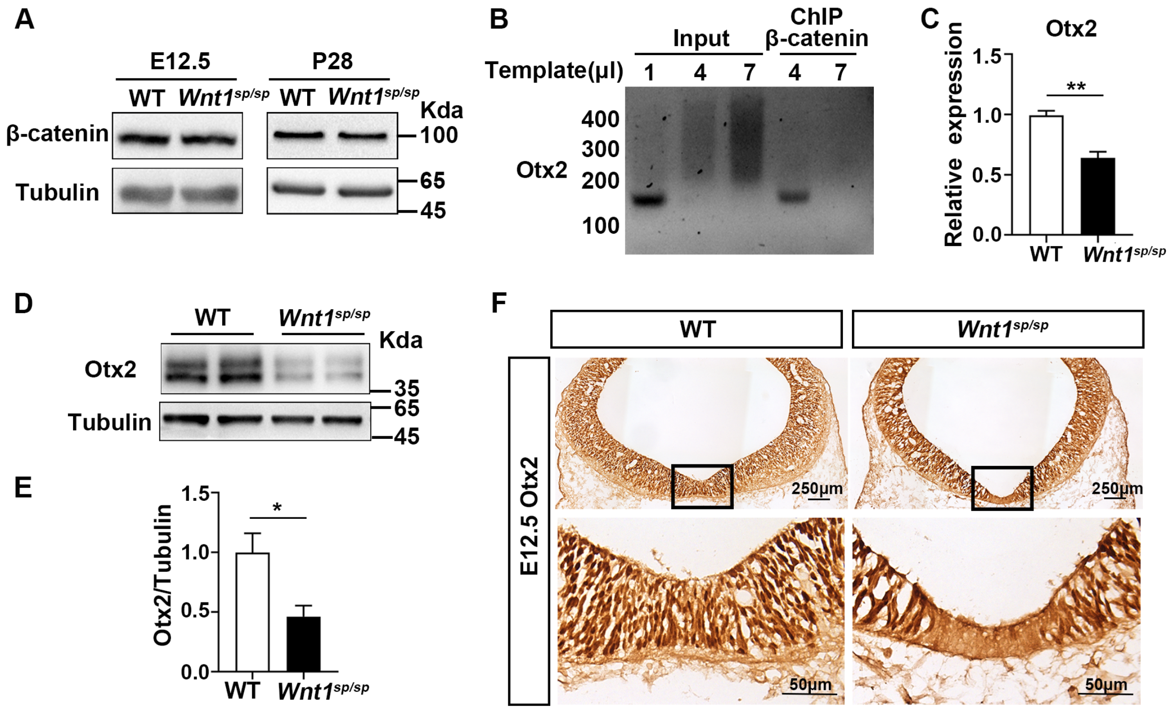

Fig. S12. *Wnt1*$^{sp/sp}$ mutation leads to an inactivation of WNT/ ß-catenin signaling pathway in vivo. E12.5 midbrain tissue was used for the following ChIP, RT-qPCR and Western blot. A. Western blot detected the expression level of ß-catenin in the brain of rats at E12.5 or P28. B. Co-IP of ß-catenin with *Otx2* in brain tissues from rats at E12.5. C. RT-qPCR detected the expression level of *Otx2* in the brain tissues of rats at E12.5. D-E. Western blot detected the expression level of Otx2 in the brain tissues of rats at E12.5 and the corresponding densitometric analysis (E) ($n = 5$ in each group). F. Immunohistochemical staining of Otx2 on brain sections in WT ($n = 3$) and *Wnt1*$^{sp/sp}$ rats ($n = 6$) at E12.5. Data are expressed as mean ± SEM. *, $p < 0.05$; **, $p < 0.01$. All Student's *t*-test.

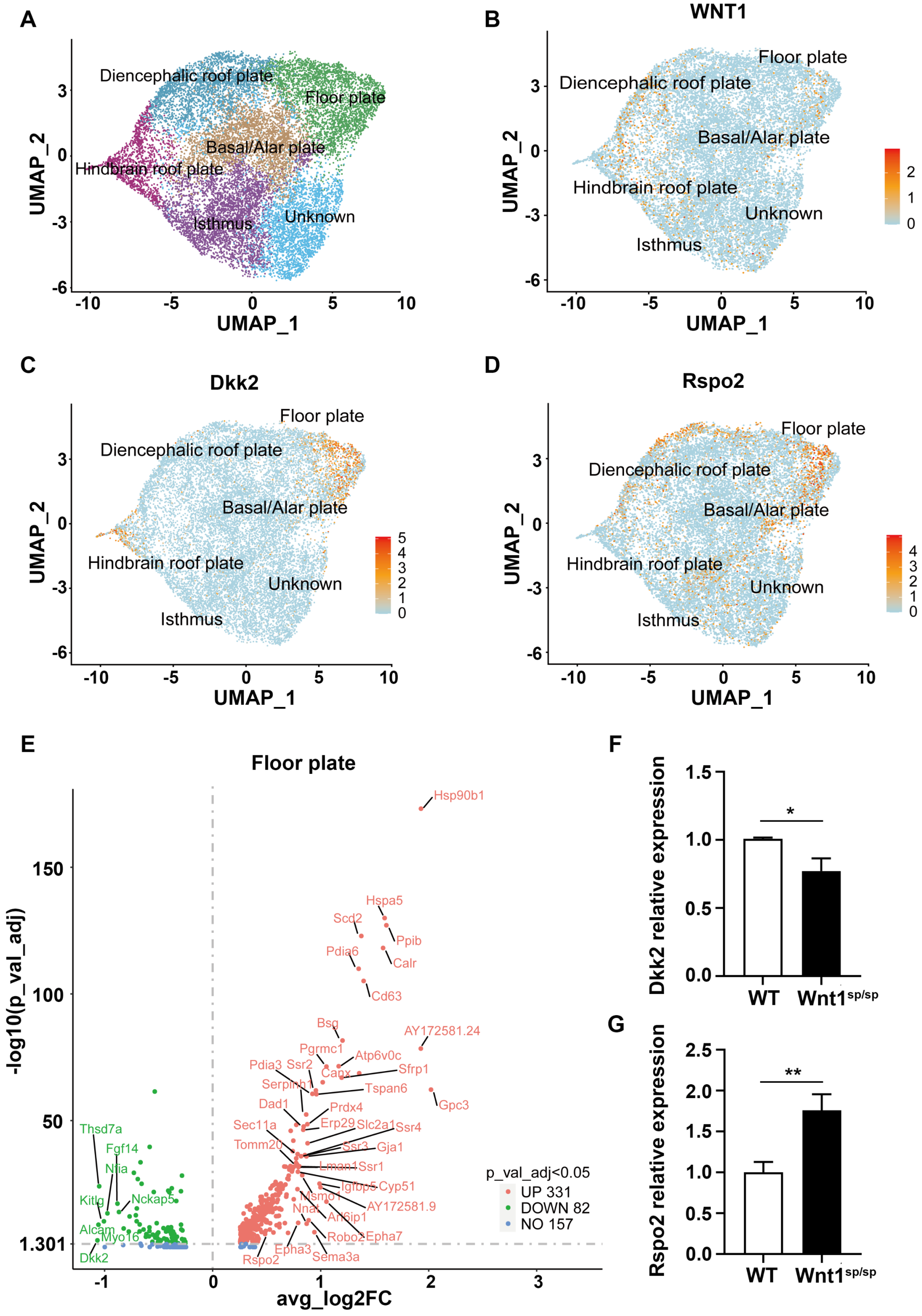

396 **Fig. S13. Compensatory changes of upstream molecules of WNT signaling pathway in**

***Wnt1$^{sp/sp}$* rats.** A. UMAP plot of neural tube organizer cluster; B. The feature plot of WNT1. The dots in orange represent WNT1^{+} cells. C. The feature plot of Dkk2. The dots in orange represent Dkk2^{+} cells. D. The feature plot of Rspo2. The dots in orange represent Rspo2^{+} cells. E. The volcano map of differential expression of genes including down-regulation (green), up-regulation (pink), or no change (blue) in the FP cluster. F. The expression level of Dkk2 (n = 4) detected by RT-qPCR in E12.5 brain tissue. G. The expression level of Rspo2 (n = 4) detected by RT-qPCR in E12.5 brain tissue. Data are expressed as mean ± SEM. *, $p < 0.05$; **, $p < 0.01$. All Student's *t*-test.

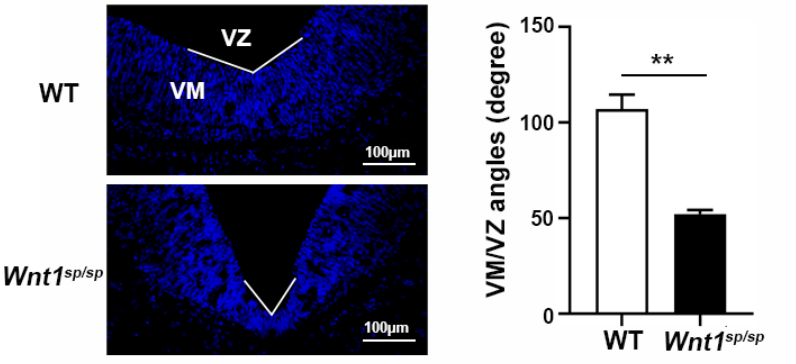

Fig. S14. Angle between VM and VZ in the midbrain floor plate. A. Nuclear staining on the midbrain coronal sections in WT (n = 3) and *Wnt1$^{sp/sp}$* rats (n = 6) at E12.5. The angle between the VM and VZ was shown in white lines; B. Quantitative analyses of the angle between the VM and VZ. VM: ventral mantle; VZ: ventricular zone. Data are expressed as mean ± SEM. ** $p < 0.01$. All Student's *t*-test.

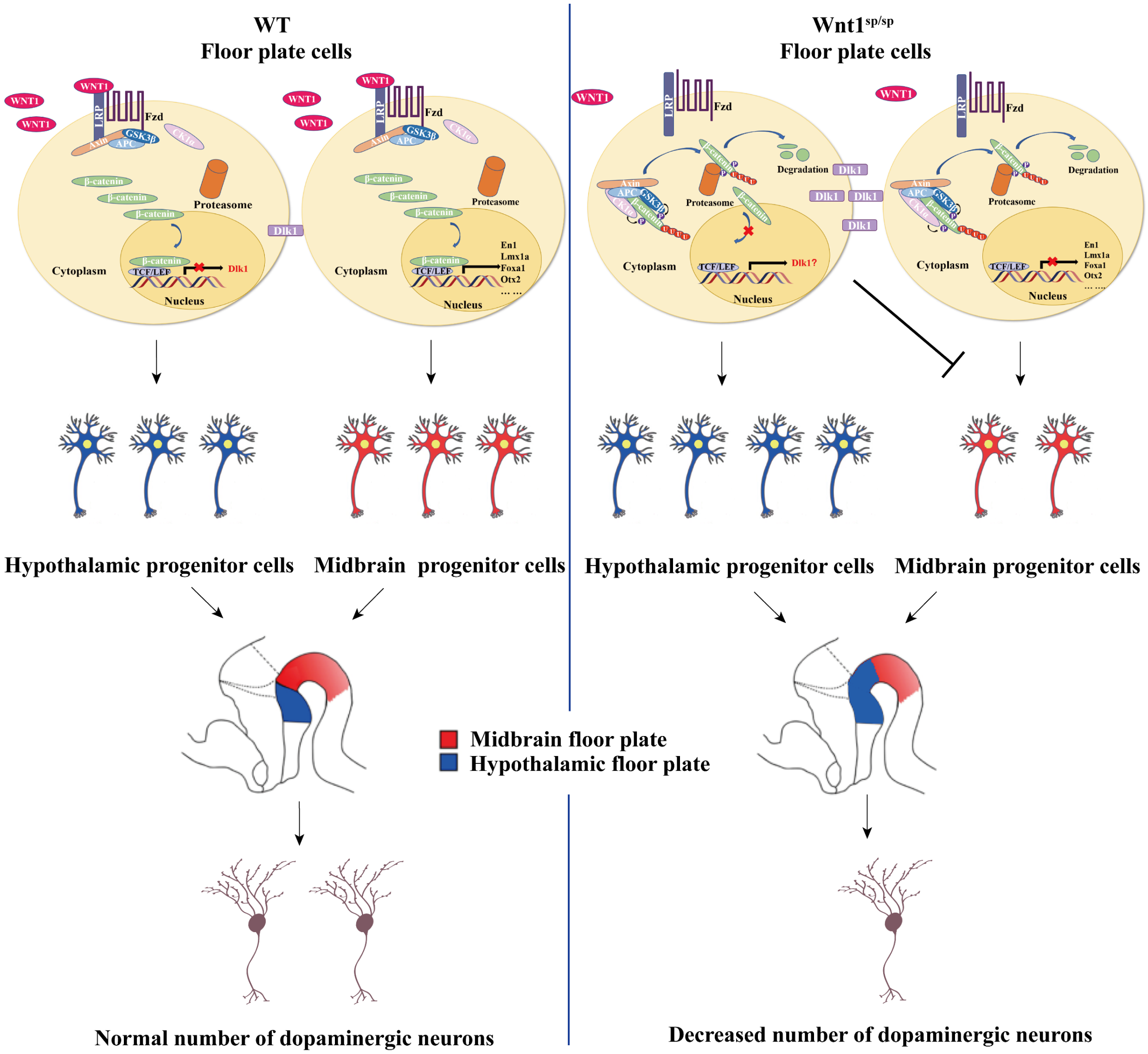

Fig. S15. The underlying pathogenic mechanism associated with *Wnt1*$^{sp/sp}$ mutation. In the normal condition, by binding to the LRP and Fzd receptor, WNT1 prevents ß-catenin from being degraded via the "destruction complex", which consists of Axin, APC, CK1α, GSK3β. The increased ß-catenin is then translocated into the nucleus from cytoplasm, and activates the expression of midbrain-related marker genes, such as En1, Lmx1a, Foxa1, and Otx2, but suppresses the expression of hypothalamic relative marker genes, such as Dlk1. The number between MHFP progenitor cells and ZHFP progenitor cells is in a balance (the picture of floor

plate is from(7)), and thus, a normal boundary between them is formed. Ultimately, the dopaminergic neurons which differentiated from the MHFP progenitor cells are developed normally. In $Wnt1^{sp/sp}$ condition, however, the expression of WNT1 is reduced, which in turn leads to the degradation of ß-catenin. Consequently, the expression of midbrain-related marker genes is suppressed while the expression of hypothalamic-related marker genes is activated. At the same time, the cells with an increased Dlk1 expression level may inhibit the neighbor cells to be specialized into midbrain floor plate progenitor cells. Accordingly, the number of the midbrain progenitor cells is decreased, and the number of the hypothalamic progenitor cells is increased. The boundary between them shifts to the side of midbrain. Finally, the mature dopaminergic neurons are dramatically reduced, leading to the occurrence of ASD.

Supplementary Tables

Supplementary Table1. Sequences used for CRISPR/Cas9 editing and primers used for genotyping of the mutant site.

gRNA	5'-ACC AGC TCA CTT ACC ACC ATC GG-3'
Donor oligo sequence	TTGCTACTGGCACTGACCGCTCTGCCCGCA<br>GCCCTGGGCGCCAACAGTGGCCGATGGTGA<br>TAAGTGAGCTGGTACGGGGTCGCCACTTGT<br>CCTGGGGCAAAGAGCCAGGAACGGGCCCTA
PCR genotyping-F	5'-CGC AAC TAT AAG AGG CGG TGC CT-3'
PCR genotyping-R	5'-CGC AGC GAG GTT AAG ACC TGT TG-3'
DNA sequencing primer	5'-GAC AGC GAA CCA TGC TGC CT-3'

Supplementary Table 2. Primers used in plasmids construction

Kpn1-EGFP-F	5'- GGT ACC ATG GTG AGC AAG GGC GAG G-3'
EGFP-Bgl2-R	5'- AGA TCT CTT GTA CAG CTC GTC CAT-3'
ScaI-Wnt1-F	5'-AGT ACT ATG GGG CTC TGG GCG CTG-3'
Wnt1-XbaI-R	5'- TCT AGA CAG ACA CTC GTG CAG TAC-3'

Supplementary Table 3. Primers used in 5'RACE

rat *Wnt1*-specific primer 1	5'-CCC TGC CTC GTT AT TGAG-3'
rat *Wnt1*-specific primer 2	5'-CGA AAT CGA TGT TGT CGC T-3'
rat *Wnt1*-specific primer 3	5'-GTG ATT GCG AAG ATA AAC G-3'
human rat *Wnt1*-specific primer 1	5'-GCC TGC CTC GTT GTT GTG AAG-3'
human rat *Wnt1*-specific primer 2	5'-GCG GAG GTG AT AGC GAA GAT A-3'
human rat *Wnt1*-specific primer 3	5'-GCC TCG GTT GAC GAT CTT GC-3'

Supplementary Table 4. Primer sequences used in RT-qPCR

Gene	Sense primer	Antisense primer
Otx2	5’-TTT TCA AGC GTC CAA TGC GG-3’	5’-AAC CAC AC TAG GCA GAG TCG-3’
Arhgef28	5’-AGC TCT CTG CGT GTC ATC TTT-3’	5’-ATA GTT CAG CCC TGG GGG AG-3’
Gprc5c	5’-GGA GAG GCC AGA CTC GGA AT-3’	5’-AGA TCA CGA GGG AAT GCC AC-3’
Trabd2b	5’-CAA TGT TCT GGG CTC CAA CTG-3’	5’-AAC CCC GAT ACA GGA AGA TGC-3’
Samd5	5’-ATC CAC TTC GCT GCT AGA CG-3’	5’-GGT GGA GAG AAG AAA CGG CA-3’
Dkk2	5’-GCA TGG TCT GTC GGA GGA AA-3’	5’-GCG AGC ACA ACA AAA CCC AT-3’
Myo16	5’-ATG ACT ACC CAG GAC CTC CC-3’	5’-GCA GGG TGG CCC TAA GAT TT-3’
Mme	5’-CAC AAA CTC TGG GGT GAG CAT-3’	5’-ACC TGA AGA ACA AGG ACG CA-3’
Aldh1a1	5’-CCC TCT GTG ACC CCT TGA AC-3’	5’-TGC CAT AAT CCT AGT TGA TTC CCA-3’
Foxa1	5’-GCA CAA TTT TCC CCG GTT CA-3’	5’-TGC CAC GGG ACT AGA ATG TG-3’
Ferd3l	5’-TTG AGG ACC AAA CAC TGG GG-3’	5’-TCT CAT AAG CGA AGG TGG GC-3’
En1	5’-CAG GAC AAA GAC GAG AGC GA-3’	5’-GTC CAC TCG GAG GAT TGC TT-3’
Lmx1a	5’-CAC CTC ACA TTT CCC TTG GC-3’	5’-CCA ACA TGT TCG GGT TGA GC-3’
Dlk1	5’-CTC ACA GCT CCC TCT ATG CG-3’	5’-TGT CAC ACA GCA ACA CGA GA-3’
Nkx2-1	5’-CCC TGG CCC CTC ACT TTT TA-3’	5’-AGG GTT TTA ATC AGA AAA AGC ATC T-3’
Fezf2	5’-ATC AAG CCG CAG GTC ATC AA-3’	5’-ATG GGG ATA GGA AGC TGG GT-3’
Lhx5	5’-GCG CGT GGC ATA TCA AAT GT-3’	5’-AGG TGG AAG ACT TTG CTC CG-3’
Wnt8b	5’-GGC TGT GAT GAC TCC CGA AA-3’	5’-AGC CTC GTT GTT GTG CAG AT-3’
Lhx2	5’-AGA CTA CTA CAG GCG GTT CTC T-3’	5’-GAG CCC AAT CCT GCA CTC TT-3’
Wnt7b	5’-TGA AGC TCG GAG CAT TGT CAT-3’	5’-ACT CCC TAC TCG GAG CTC TTG-3’
Fezf1	5’-CAA AAT GCC TGC TGA CCG TT-3’	5’-GAA TCA ACT CCG ACG TGC AA-3’
Nr2e1	5’-CGG ATC AAC AAG CCG CAT TT-3’	5’-CTT GTC TAC GGG GCA TCC TC-3’
Six3	5’-GTT GCG GGC AGA AAG CAT AA-3’	5’-AAA TCG TCA TGC AGG TGG GG-3’
Emx2	5’-CGA CTC CGT TCC ATT CTG GG-3’	5’-GGT CGC TAT TAC TCG CCC TG-3’
Arx	5’-CTC AGC ACC ACT CAA GAC CA-3’	5’-GGA CAG GGA CAA GGG CAA AT-3’
Gapdh	5’-ACA GCA ACA GGG TGG TGG AC-3’	5’-TTT GAG GGT GCA GCG AAC TT-3’
Dkk2	5’-GCA TGG TCT GTC GGA GGA AA-3’	5’-GCG AGC ACA ACA AAA CCC AT-3’
Rspo2	5’-CTG TCC AGG AGG TGG GTC TA-3’	5’-GCA TTT ATT GTG TGG AGG AGG G-3’

References:

1. Xian C, Zhu M, Nong T, Li Y, Xie X, Li X, et al. A novel mutation in ext2 caused hereditary multiple exostoses through reducing the synthesis of heparan sulfate. Genetics and molecular biology. 2021;44(2):e20200334.

2. Jiao J, Nakajima A, Janssen WG, Bindokas VP, Xiong X, Morrison JH, et al. Expression of NR2B in cerebellar granule cells specifically facilitates effect of motor training on motor learning. PLoS One. 2008;3(2):e1684.

3. Chartoff EH, Marck BT, Matsumoto AM, Dorsa DM, Palmiter RD. Induction of stereotypy in dopamine-deficient mice requires striatal D1 receptor activation. Proceedings of the National Academy of Sciences of the United States of America. 2001;98(18):10451-6.

4. Chen Q, Nakajima A, Choi SH, Xiong X, Sisodia SS, Tang YP. Adult neurogenesis is functionally associated with AD-like neurodegeneration. Neurobiol Dis. 2008;29(2):316-26.

5. Wang X, Lu J, Xie W, Lu X, Liang Y, Li M, et al. Maternal diabetes induces autism-like behavior by hyperglycemia-mediated persistent oxidative stress and suppression of superoxide dismutase 2. Proceedings of the National Academy of Sciences of the United States of America. 2019;116(47):23743-52.

6. Chen Q, Tang M, Mamiya T, Im HI, Xiong X, Joseph A, et al. Bi-directional effect of cholecystokinin receptor-2 overexpression on stress-triggered fear memory and anxiety in the mouse. PLoS One. 2010;5(12):e15999.

7. Nouri N, Awatramani R. A novel floor plate boundary defined by adjacent En1 and Dbx1 microdomains distinguishes midbrain dopamine and hypothalamic neurons. Development (Cambridge, England). 2017;144(5):916-27.
